# SUMO conjugation susceptibility of Akt/protein kinase B affects the expression of the pluripotency transcription factor Nanog in embryonic stem cells

**DOI:** 10.1371/journal.pone.0254447

**Published:** 2021-07-09

**Authors:** Marcos Francia, Martin Stortz, Camila Vazquez Echegaray, Camila Oses, Paula Verneri, María Victoria Petrone, Ayelen Toro, Ariel Waisman, Santiago Miriuka, María Soledad Cosentino, Valeria Levi, Alejandra Guberman

**Affiliations:** 1 Instituto de Química Biológica de la Facultad de Ciencias Exactas y Naturales (IQUIBICEN, CONICET-UBA), Departamento de Química Biológica, Facultad de Ciencias Exactas y Naturales, Universidad de Buenos Aires, Ciudad Autónoma de Buenos Aires, Argentina; 2 Laboratorio de Investigación Aplicada a las Neurociencias Fundación para la Lucha contra las Enfermedades Neurológicas de la Infancia (LIAN, FLENI-CONICET), Escobar, Provincia de Buenos Aires, Argentina; 3 Departamento de Fisiología y Biología Molecular y Celular, Facultad de Ciencias Exactas y Naturales, Universidad de Buenos Aires, Ciudad Autónoma de Buenos Aires, Argentina; Manipal Institute of Regenerative Medicine, INDIA

## Abstract

Akt/PKB is a kinase involved in the regulation of a wide variety of cell processes. Its activity is modulated by diverse post-translational modifications (PTMs). Particularly, conjugation of the small ubiquitin-related modifier (SUMO) to this kinase impacts on multiple cellular functions, such as proliferation and splicing. In embryonic stem (ES) cells, this kinase is key for pluripotency maintenance. Among other functions, Akt is known to promote the expression of Nanog, a central pluripotency transcription factor (TF). However, the relevance of this specific PTM of Akt has not been previously analyzed in this context. In this work, we study the effect of Akt1 variants with differential SUMOylation susceptibility on the expression of Nanog. Our results demonstrate that both, the Akt1 capability of being modified by SUMO conjugation and a functional SUMO conjugase activity are required to induce Nanog gene expression. Likewise, we found that the common oncogenic E17K Akt1 mutant affected Nanog expression in ES cells also in a SUMOylatability dependent manner. Interestingly, this outcome takes places in ES cells but not in a non-pluripotent heterologous system, suggesting the presence of a crucial factor for this induction in ES cells. Remarkably, the two major candidate factors to mediate this induction, GSK3-β and Tbx3, are non-essential players of this effect, suggesting a complex mechanism probably involving non-canonical pathways. Furthermore, we found that Akt1 subcellular distribution does not depend on its SUMOylatability, indicating that Akt localization has no influence on the effect on Nanog, and that besides the membrane localization of E17K Akt mutant, SUMOylation is also required for its hyperactivity. Our results highlight the impact of SUMO conjugation in the function of a kinase relevant for a plethora of cellular processes, including the control of a key pluripotency TF.

## Introduction

Embryonic stem (ES) cells are pluripotent cells derived from the inner cell mass (ICM) of mammalian blastocysts [1, 2]. Under specific culture conditions, they can indefinitely self-renew and preserve their potential to differentiate to derivatives of all the three germ layers [3]. The cytokine Leukemia Inhibitory Factor (LIF), along with its receptor, constitutes the starting point of several signal transduction cascades that play a crucial role in maintaining the pluripotent state of mouse ES cells. In these cells, LIF activates three signaling pathways: JAK/STAT3, PI3K/Akt and MEK/ERK. While the last one promotes mouse ES cells differentiation, the others facilitate their self-renewal ultimately inducing the expression of the fundamental transcription factors (TFs) Oct4, Sox2 and Nanog [4]. These TFs constitute the core network of pluripotency promoting the expression of key pluripotency genes and repressing genes associated with cell differentiation [5, 6]. Specifically, Nanog has a central role in pluripotency of both cells from the ICM and ES cells; and sustains self-renewal in ES cells in the absence of LIF [7, 8]. Nanog gene regulation is exerted by the balance of multiple TFs binding to its regulatory region, highly dependent on the cell context (reviewed in [9]).

Particularly, Nanog transcription is induced through the activation of PI3K/Akt signaling pathway by LIF followed by phosphorylation and the subsequent inactivation of GSK3-β [10]; and also through Akt induction of Tbx3 [11].

Akt, also known as Protein Kinase B, is a serine/threonine kinase involved in a wide variety of cellular processes and its deregulation is associated to several human diseases [12]. Multiple post-translational modifications (PTMs), such as phosphorylation in several residues, acetylation or peptide conjugation, have been reported to regulate Akt activity and substrate specificity thus modulating particular cellular responses [13]. Akt activation is downstream of the LIF signaling pathway [4] in mouse ES cells, and it occurs as a consequence of the highly characterized events of phosphorylation at T308 by PDK1 and S473 by mTORC2 [14]. Akt signaling maintains ES cell pluripotency and regulates stemness in several stem cell systems, like mouse and primate ES cells [15].

SUMO (small ubiquitin-related modifier) is a small peptide from the ubiquitin family that can be covalently attached to different target proteins modifying their activity, structure, sub-cellular localization and interactions with protein partners or nucleic acids [16]. This reversible and transient PTM, known as SUMO conjugation or “SUMOylation”, involves the activation and ultimate ligation of the SUMO peptide to lysine residues by the SUMO conjugase enzyme Ubc9 [17]. This process can be reversed by deconjugating SUMO protease enzymes, making SUMOylation a dynamic process that depends on the cell context [18]. Particularly, it has been reported that modification of Akt by SUMO conjugation regulates the activity of this kinase with direct consequences in splicing patterns, cell growth, survival and oncogenic potential of cultured cell lines [19–22]. However, the relevance of this specific PTM of Akt in ES cells had yet to be explored.

Based on the widely known relevance of Akt in ES cells, and the gap in our understanding of how signaling pathways interact with the intrinsic network of pluripotency TFs, we aimed to explore the impact of Akt SUMOylatability on Nanog gene regulation. Here, we show that the SUMO-conjugation susceptibility of Akt impacts on the previously reported inductor effect of this kinase on Nanog gene expression, evidencing a novel link between this Akt PTM and a key pluripotency TF in ES cells.

## Materials and methods

### Cell culture

W4 ES cell line was provided by the Rockefeller University Core Facility. Cells were routinely cultured in ES cell medium (referred as 2i + LIF medium within the manuscript) containing DMEM, 2 mM Glutamax, 100 mM MEM NEAA, 0.1 mM 2-mercaptoethanol, 100 U/ml penicillin, 100 mg/ml streptomycin and 15% FBS (Gibco), LIF and the two-inhibitors (2i) cocktail consisting of 1 μM PD0325901 (Tocris) and 3 μM CHIR 99021 (Tocris). Cells were plated on 0.1% gelatin coated dishes at 37°C in a 5% CO_2_ (v/v) incubator and passaged every three days. All experiments were performed in these conditions unless it is indicated otherwise. For the experiment in S2 Fig, NIH/3T3 cells (ATCC) were cultured as previously reported [23].

### Transfection and luciferase activity assay

**Fig 1:**
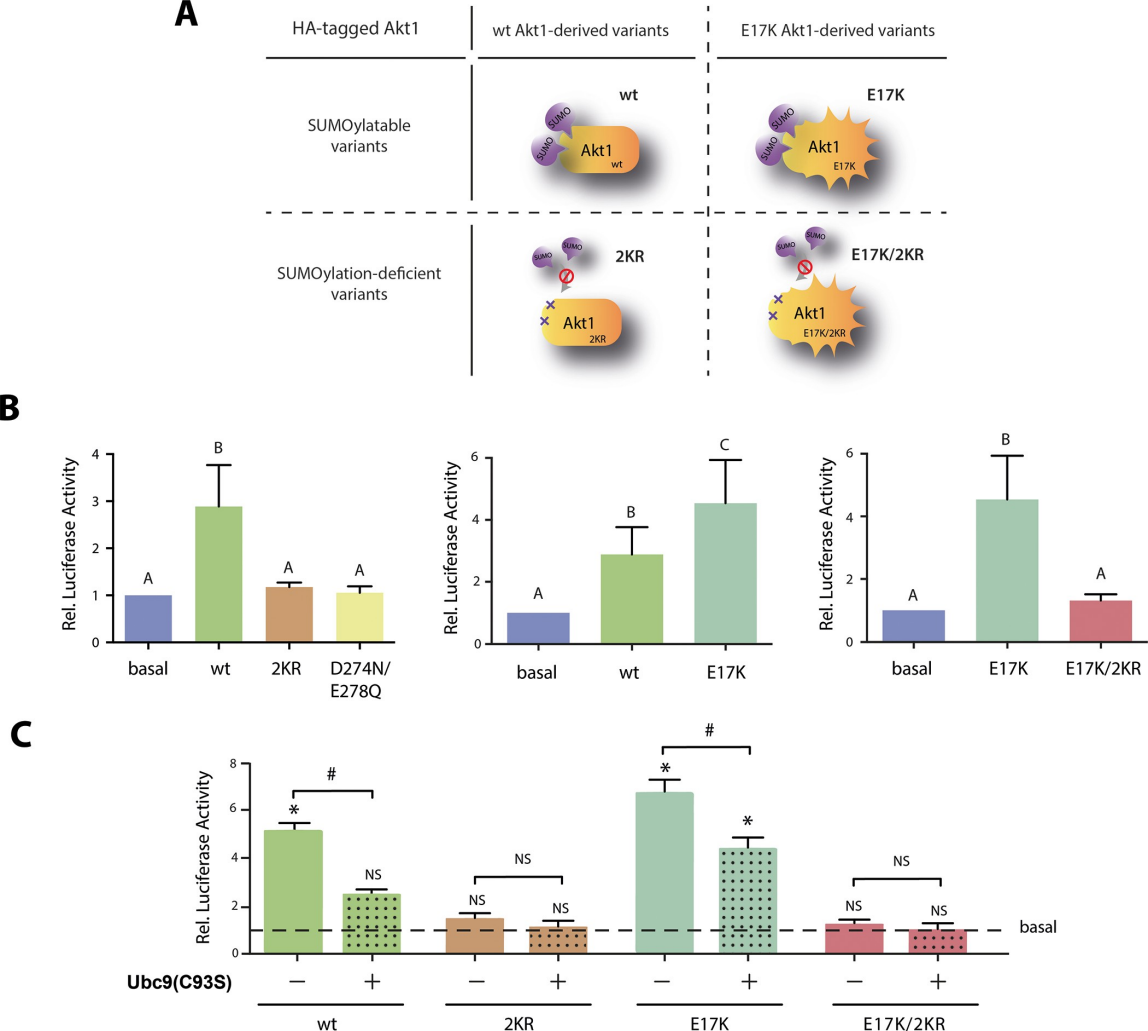
SUMO conjugation of Akt impacts on Nanog promoter activity in ES cells. (A) Cartoon representing the main features of the Akt variants most used in this work. SUMO group is represented by violet balloons, hyperactivity-inducing E17K mutation is depicted with thorny edges and mutated lysines in 2KR and E17K/2KR variants are indicated by the Xs. (B) ES cells were transfected with Nanog5P reporter along with an expression vector encoding for either wt Akt1, the Akt1 mutants 2KR, D274N/E278Q, E17K, E17K/2KR or the empty vector (basal). Luciferase activity was measured in extracts obtained from transfected cells maintained in standard medium in the presence of LIF and 2i for 72 h. Results were referred to the control condition (basal) and are shown as mean ± SEM of three independent experiments. Statistical analysis was performed by linear mixed models (LMM) with a randomized block design (RBD). Significant differences were assessed using the DGC test. Different letters indicate significant differences among cellular conditions (p< 0.05). (C) ES cells were transfected with Nanog5P reporter along with the wt, 2KR, E17K and E17K/2KR Akt1 variants or the empty vector (basal, solid bars) and also including (+) or not (-) a vector encoding the dominant negative Ubc9(C93S) (dot-patterned bars), as indicated. Results were referred to the corresponding control condition (basal, indicated as a dashed line) and are shown as fold change mean ± SEM of three independent experiments. Statistical analysis among groups was performed by lineal mixed models (LMM) with a randomized block design (RBD) and comparisons were performed using the Tukey’s HSD test. Fold change comparisons between groups were assessed by two-tailed paired *t* test. Asterisks indicate significant differences and NS above bars denote no significant differences of each condition compared to the basal (p< 0.05). Hashes indicate significant differences between fold change of the same Akt1 variant with (+) or without (-) Ubc9(C93S) (p<0.05).

For the luciferase activity assay, ES cells were plated in a 24-well plate (36000cells/well) using ES cell medium. For the experiment in S2 Fig, NIH/3T3 cells were plated in a 24-well plate (17000 cells/well) as previously reported [23–25]. After 24 h, medium was replaced for the culture medium according to the specified conditions of each experiment and cells were co-transfected with 650 ng of each Akt1 variant vector [19] along with 600 ng of Nanog5P reporter. This Luciferase reporter is a kind gift from Austin Cooney (Addgene plasmid # 16337) [26]. In experiment from Fig 1C, 500 ng of Ubc9(C93S) [27] vector were additionally co-transfected. Transfection was carried out using PEI (Linear Polyethylenimine 25 kDa, Polysciences Inc.) with a DNA/PEI ratio of 1:3. After overnight incubation, medium was replaced again, and 48 h later cells were lysed and assayed for luciferase activity using the Dual Luciferase kit (Promega) on a GloMax Multi Detection System (Promega). Total protein mass was measured by Bradford protocol and used for normalization in each transfection assay. Experiments were performed in triplicate and repeated at least three times.

### Immunostaining

Immunofluorescence was performed as previously described [28, 29] with minor modifications: cells were fixed by treatment with 4% paraformaldehyde for 15 minutes and permeabilized with 0.1% Triton X-100 in PBS for 10 minutes. Blocking was performed with 1% normal goat serum (Sigma) in PBS-Tween 0.1% solution for 1 hour. Primary antibodies in blocking solution were added to the samples that were incubated at room temperature for 1 hour and washed three times in PBS-Tween 0.05% for 5 minutes. Secondary antibodies solutions were prepared in blocking solution including DAPI (Sigma) and were incubated with the samples at room temperature for 1 hour. Samples were washed as described above and imaged in an Olympus IX71 or FV1000 microscope. All the antibodies used are listed in S1 Table.

### Western blot analysis

Western blot was performed as previously described [28, 29] with minor modifications: proteins were collected from cell lysates with RIPA buffer, run in 12% SDS-polyacrylamide gel electrophoresis and transferred to PVDF membranes (Amersham). Membranes were blocked for 1 hour at room temperature in 0.1% Tween-20 Tris-Buffered Saline solution (TBST) containing 5% nonfat dry milk. Primary antibodies were incubated overnight at 4°C in blocking solution. Secondary antibodies were incubated at room temperature for 1 hour. Membranes were washed three times with TBST and were revealed with ECL Prime Western Blotting Detection (GE Healthcare) in an Amersham imager 600 (GE Healthcare). All the antibodies used are listed in S1 Table.

### Construction of Tbx3 knockout ES cell line

The Tbx3 knockout ES cell line was generated using CRISPR/Cas9 technology on W4 ES cell line as previously reported [28, 29]. PSpCas9(BB)-2A-Puro vectors containing sgRNA for CRISPR guided Tbx3 knockout were designed and kindly supplied by Dr. Judith Davie [30]. Sequences for sgRNA are listed in S2 Table. Briefly, wild type W4 ES cells were transfected with both plasmids using Linear Polyethylenimine (PEI, Polysciences) with a DNA/PEI ratio of 1:5 and selected with 3 μg/ml puromycin from 24 to 72 h post-transfection. After limiting dilution, multiple individual clonal ES cell lines were obtained. Tbx3 knockout was analyzed and confirmed by western blotting and immunostaining against Tbx3 protein (S4A Fig).

### Confocal imaging

As previously detailed [28, 31], confocal images were acquired in a FV1000 laser scanning microscope (Olympus) using an Olympus UPlanSApo 60x oil immersion objective (NA = 1.35). Cy3 were excited using a He-Ne green laser at 543 nm (average power at the sample, 700 nW). Fluorescence was detected with a photomultiplier set in the pseudo photon-counting detection mode, using 560–660 nm filtering for Cy3 detection. An averaged image was obtained from 10 consecutive images acquired per cell.

### Bioinformatics analysis

The Stemformatics web tool (https://www.stemformatics.org) [32, 33] was used to evaluate Nanog gene expression (from LC-MS and RNAseq experiments) and epigenetic marks on its promoter (from H3K4me3 and H3K27me3 ChIP-seq, and whole genome bisulfite sequencing experiments), in both ES cells and MEFs from publicly available datasets for S2B Fig. Data normalization, transformation and annotation methods are available at Stemformatics documentation (https://www.stemformatics.org/Stemformatics_data_methods.pdf). The ChIP-Atlas database (http://chip-atlas.org) [34] was used to evaluate the enrichment profile of Tbx3 in the 2.5 Kbp region of the Nanog genomic locus included in Nanog5P reporter in S4C Fig. The results shown correspond to the analysis of public ChIP-seq data from experiments performed in ES cells. Data was visualized using the Integrative Genomics Viewer (IGV) software [35]. Data normalization, transformation and annotation methods are available at ChIP-Atlas documentation (https://github.com/inutano/chip-atlas/wiki). Full meta-data of all analyzed datasets is available at S3 Table.

### Statistical analysis

Data was shown and analyzed as previously described [28, 31]. Experimental results were expressed as mean ± standard error of the mean (SEM) of at least three biological replicates. In general, statistical significance between groups was analyzed using randomized block design (RBD) ANOVA. Residuals fitted normal distribution and homogeneity of variance. Otherwise, transformation of data (log) was applied in some cases to meet both assumptions.

Additionally, post-hoc multiple comparisons between means were assessed using the Tukey’s HSD test for experiments with equal sample size, or DGC [36] test when treatments presented unequal sample sizes. Differences were regarded as significant at least with a p-value of ≤0.05. We used letters to indicate significant differences when comparisons were performed among multiple treatments. While different letters indicate significant differences, identical letters correspond to non-significant differences between treatments. Contrary, when each sample was only compared to the control condition, we used asterisks. In some experiments, significance between groups was analyzed by linear mixed models (LMM) due to imbalance of data. Specific information about analysis is presented in each figure legend. All the statistical analysis was performed using Infostat Software [37]. Data in Fig 4C was analyzed using the Marascuilo’s test [38], for comparing multiple proportions.

## Results

### Akt SUMOylation induces Nanog promoter activity in ES cells

To explore a possible involvement of Akt SUMOylation in Nanog gene regulation, we first evaluated the effect of Akt1 variants with different capacities of being SUMOylated on the Nanog promoter activity in ES cells. These variants are schematized in Fig 1A and described below. Also, we took advantage of a luciferase gene reporter driven by a 2.5 Kbp fragment of the promoter region from mouse Nanog genomic *locus* (Nanog5P) [26]. This reporter has been previously used in different cellular contexts and evidenced to be a good model to study the endogenous gene [26, 39–41]. We co-transfected this reporter with each of the expression vectors encoding the different Akt1 variants and evaluated their effect by measuring the luciferase activity. We first verified by immunofluorescence (IF) that the proportion of cells transfected with the different Akt1 variants was similar (S1A Fig), and that they showed comparable expression levels assessed by Western blot (S1B Fig). No substantial differences were detected.

Then, we evaluated the effect of wild type Akt1 (wt) and a SUMOylation-deficient mutant in which lysines 276 (K276) and 301 (K301) were replaced by arginines, Akt1 2KR [19]. As expected, wt Akt1 induced the Nanog promoter producing a significant increase in luciferase activity, in agreement with the previously reported Nanog induction by Akt [11]. In contrast, luciferase levels determined upon over-expression of Akt1 2KR were similar to the levels determined in the basal condition in which cells were co-transfected with an empty vector. These results indicate that the 2KR mutations introduced in Akt1 completely abolished its effect on the Nanog reporter (Fig 1B, left panel). We also assayed another Akt1 mutant that, while preserving the SUMOylation target K276, it displays reduced SUMOylation due to the replacement of the negatively charged residues flanking the SUMO consensus sequence (D274N/E278Q) [19]. This mutant produced the same effect on Nanog reporter than Akt1 2KR (Fig 1B, left panel), further supporting the role of this PTM of Akt.

We next evaluated the effect of a hyperactive Akt1 mutant, Akt1 E17K. Relevantly, this mutation has been found in somatic cells of human breast, colorectal and ovarian cancer [42]. Moreover, this mutant was reported to display higher SUMO conjugation levels than the wt version [19]. According to the luciferase assay, Akt1 E17K induced Nanog reporter even to a higher extent than Akt wt (Fig 1B, middle panel). Remarkably, the Akt1 variant E17K/2KR which combines the three mutations described above (E17K/K276,301R) and shows diminished SUMOylation [19], had no effect on the transcription of Nanog reporter (Fig 1B, right panel), adding new evidence in support of the relevance of Akt SUMOylatability. Interestingly, this effect seems to be specific of ES cells, or at least not generalized to different cell types nor a bias of this reporter, since the response of Nanog5P reporter to Akt variants was completely different in NIH/3T3 cells (S2A Fig). These cells are a terminally differentiated mouse embryonic fibroblast (MEF)-derived cell line in which we have not detected Nanog expression [23] and have previously used it as a heterologous non-pluripotent system [23–25]. In order to further explore differences in Nanog gene expression between MEFs and ES cells, we performed omics analysis from publicly available data [43, 44] to investigate Nanog mRNA and protein levels, and the epigenetic status of Nanog locus in both cell types (S2B Fig). These results highlight the differences in Nanog gene status between these two cell types, since in MEF, both Nanog mRNA and protein are undetectable and its promoter region presents the repressive marks, H3K27me3 and 5-mC, while the opposite pattern is observed in ES cells, which displays high Nanog expression and the mark associated with active chromatin, H3K4me3. Interestingly, not only no induction is exerted by the different Akt1 variants on the Nanog reporter, but they even seem to repress this promoter in this non-pluripotent cell context, indicating the existence of a at least, a non-generalized mechanism by which Akt1 exerts its effect on Nanog.

Up to this point, the induction exerted by wt and E17K Akt variants in ES cells and the lack of this effect with the mutants with diminished SUMOylatability strongly suggest that this PTM of Akt is required for Nanog gene regulation in the context of pluripotent cells.

With the purpose of further exploring this, we challenged the requirement of this Akt1 PTM by interfering with the SUMO-conjugation pathway, exploiting a dominant-negative mutant of Ubc9, the enzyme responsible of SUMO conjugation to the target protein [17]. We evaluated the effect of the Akt1 variants on the Nanog promoter in the presence of Ubc9(C93S), an Ubc9 mutant that lacks SUMO conjugase activity [45] and that also impedes the activity of the endogenous protein [27]. As shown in Fig 1C, co-transfection of the dominant-negative Ubc9 impaired the inductive effect on the Nanog reporter of both Akt1 SUMOylatable variants, wt and E17K. Specifically, Ubc9(C93S) completely abolished the effect of wt Akt1 and significantly reduced the induction exerted by the E17K hyperactive mutant. As expected, no changes were observed in the results obtained with the SUMOylatability diminished Akt1 mutants, 2KR and E17K/2KR. As a whole, these results demonstrate that SUMO conjugation to Akt1 is required for the induction of Nanog promoter activity.

### Nanog protein levels are increased by SUMOylatable Akt variants

**Fig 2:**
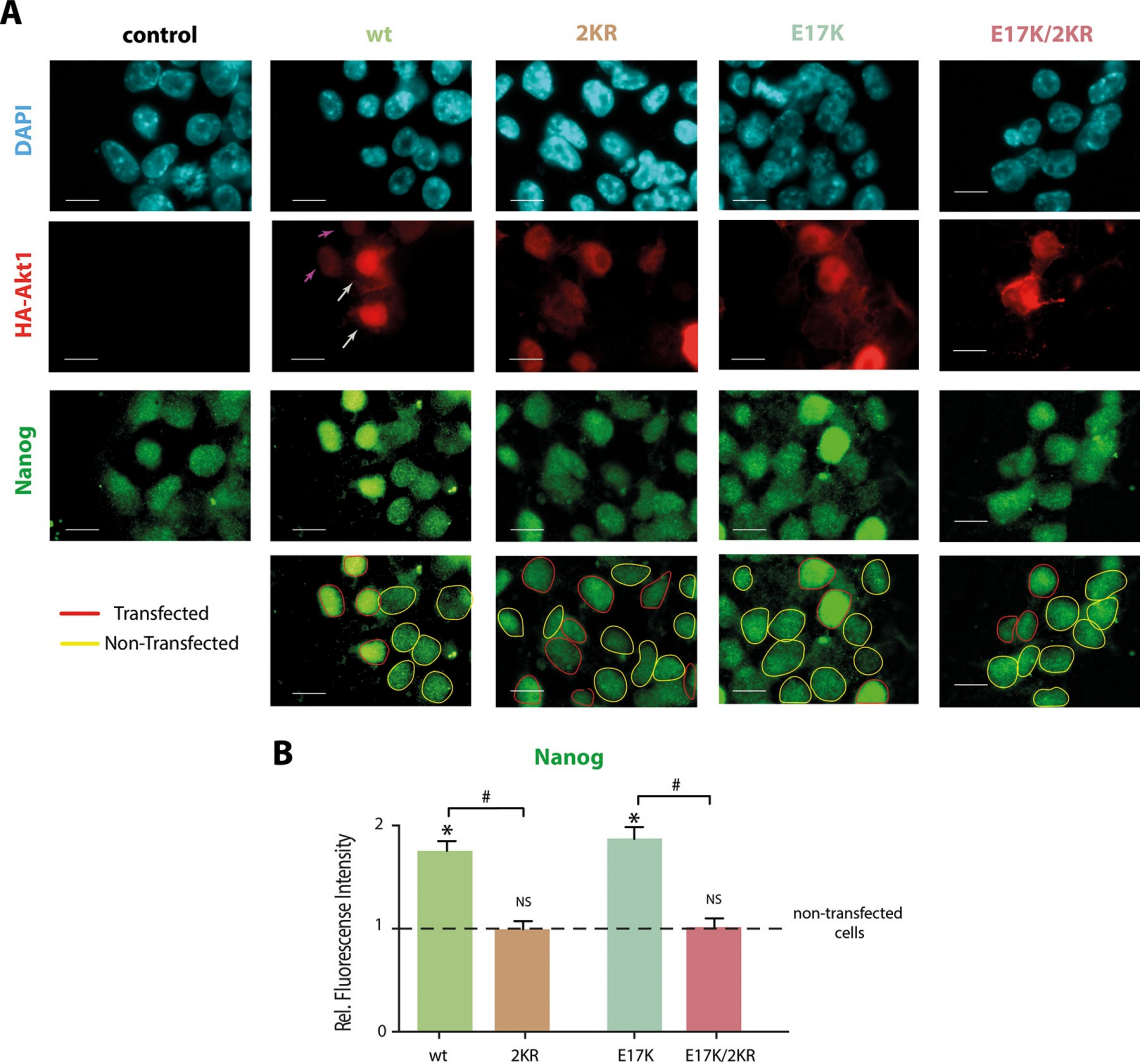
Nanog protein levels are increased by SUMOylatable Akt variants while mutants with diminished SUMOylatability have no effect.

We next studied if Akt SUMOylatability affects Nanog protein levels. For this purpose, we transfected the Akt variants described in the previous section and analyzed Nanog protein levels by IF. The fluorescence intensity of Nanog immunostaining in those transfected cells was compared to Nanog signal in non-transfected ES cells within the same field. The transfected cells were identified by IF against the HA tag included at the N-terminus of the sequence of all Akt variants utilized (Fig 2A). Fig 2B shows that both wt Akt1 and the hyperactive E17K mutant produced an increase in Nanog protein levels compared with non-transfected cells. Notoriously, 2KR and E17K/2KR mutants did not affect Nanog levels. Moreover, transfected and non-transfected cells displayed similar Nanog protein levels in the presence of these SUMOylation-impaired mutants confirming that the transfection process *per se* does not affect Nanog expression. Consistently, ES cells transfected with the empty vector (control) also presented similar levels of Nanog (Fig 2A). Noteworthy, the increase in Nanog levels seems to be independent of the amounts of HA-Akt1 expressed since we observed a similar effect on Nanog when comparing all transfected cells within the same condition. Remarkably, this can be observed even in those cells with very low HA-Akt1 levels (Fig 2A, arrows), suggesting that minimal amounts of transfected SUMOylatable variants are enough to exert its inductive effect on Nanog expression. Altogether, these results suggest that Akt SUMOylatability is required to increase endogenous Nanog protein levels.

ES cells were transfected with the indicated expression vector encoding for either wt Akt1, the Akt1 mutants 2KR, E17K, E17K/2KR or with the empty vector (control). After transfection, cells were maintained in standard ES cell medium in the presence of LIF and 2i for 72 h and then fixed for immunofluorescence analysis. Nuclei were stained with DAPI. Nanog protein and transfected Akt were visualized by immunostaining against Nanog and HA tag, respectively. Transfected cells were identified by HA positive signal. (A) Representative epifluorescence microscopy images of Nanog and HA immunostaining for cells transfected with each Akt variant. Nuclei of transfected cells are indicated with red continuous lines and nuclei of non-transfected cells with yellow discontinuous lines. Gray long arrows indicate HA-Akt1-high-expressing cells and purple short arrows, HA-Akt1-low-expressing cells. Scale bars represent 10 μm. (B) Nanog intensity signal was quantified and compared between transfected and non-transfected cells from the same field. Bars represent fluorescence intensity mean ± SEM of 350–360 cells from four independent experiments relative to the mean signal of the corresponding non-transfected cells. Asterisk indicates significant differences and NS denotes no significant differences between transfected and the corresponding non-transfected cells, evaluated using a two-tailed paired *t* test (p<0.0001). Hash indicates significant differences between relative fluorescence intensity of treatments evaluated by superposition of 95% confidence intervals of the means.

### Neither GSK3-β nor Tbx3 are essential mediators of the effect exerted by SUMOylatable Akt1

In order to delve into the molecular signaling underlying the Nanog gene regulation by Akt, we analyzed if the interference of any of the two main Akt1 mediators that ultimately affect Nanog expression in ES cells, GSK3-*β* [10] and Tbx3 [11, 46], modifies the effect on the Nanog reporter described previously. We hypothesized that the perturbation of an essential factor would interfere with the induction exerted by the SUMOylatable Akt variants. Therefore, if the induction of the Nanog reporter occurs in a condition of interference, it would be indicative that the factor interfered is dispensable. Specifically, in the case of disrupting an essential mediator, we would expect that the reporter activity in the presence of wt Akt was lower than that observed in the control condition without disruption (schematized in S3 Fig, panel i), just as we had previously observed in Fig 1C when the dominant-negative Ubc9 was co-transfected. On the contrary, if the factor evaluated is non-essential in the SUMOylatable Akt effect, the response should be similar to the control condition. S3 Fig, panel ii, schematizes the expected outputs of the experiments in those cases in which the disrupted factor is a mediator or is non-essential to the studied effect (panel iii).

**Fig 3:**
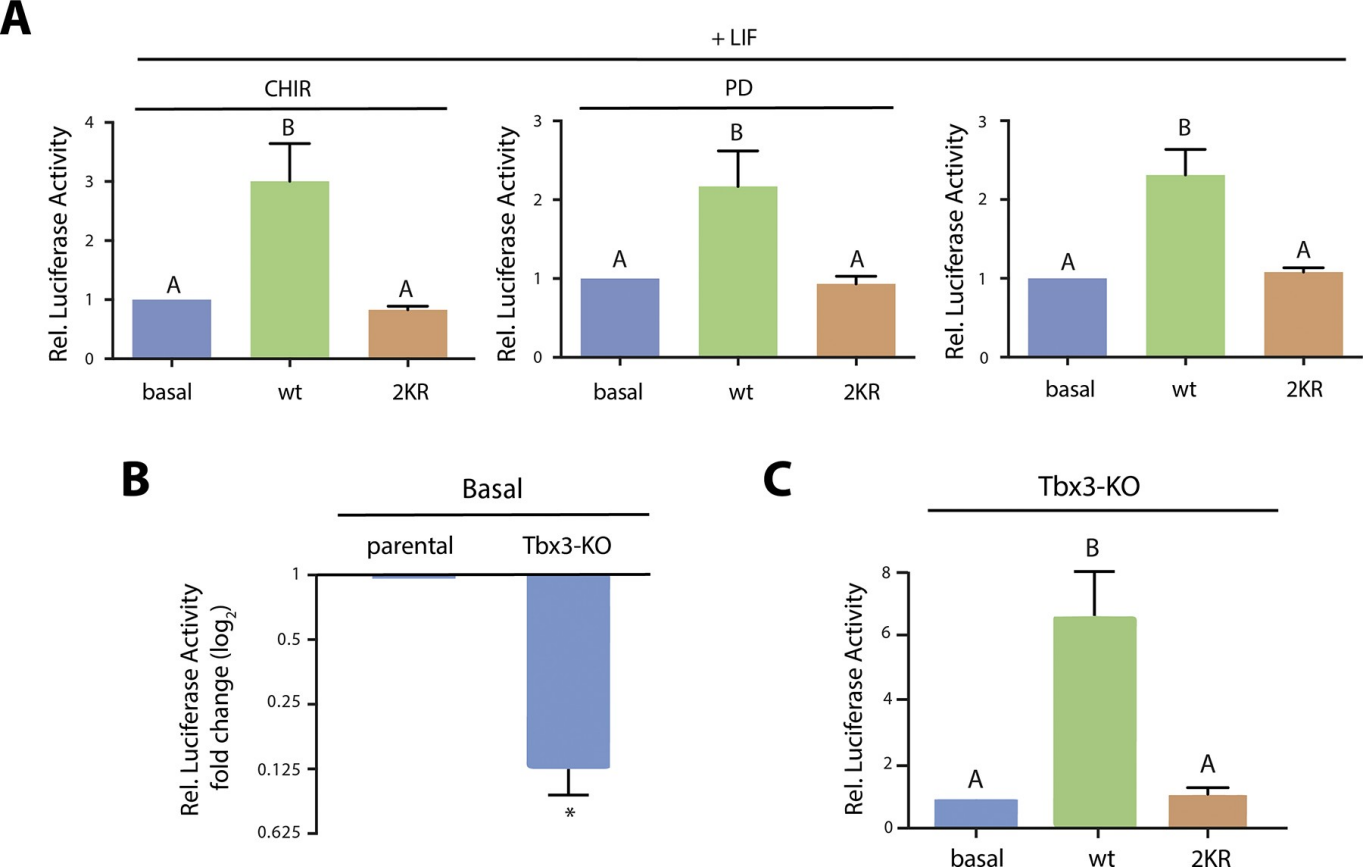
GSK3-β and Tbx3 are non-essential factors for the effect exerted by SUMOylatable Akt1. (A) ES cells were transfected with Nanog5P reporter along with an expression vector encoding for either wt Akt1, the Akt1 mutant 2KR or the empty vector (basal), as indicated under each bar. Cells were maintained in standard ES cell medium in the presence of LIF and either CHIR 99021 (CHIR) or PD 0325901 (PD), as indicated in each panel. Luciferase activity was measured in cell extracts obtained 72 h after transfection. Results were referred to the control (basal) of each condition and are shown as mean ± SEM of three independent experiments. Statistical analysis was performed by linear mixed models (LMM) with a randomized block design (RBD). Comparisons were performed using the Tukey’s HSD test. Different letters indicate significant differences between treatments (p< 0.05). (B,C) wt and Tbx3-KO ES cells were maintained in standard ES cell medium (LIF+2i). Transfection and luciferase activity measurement was performed as indicated above. Results were referred to the control condition (B: parental, C: basal) and are shown as mean ± SEM of three independent experiments. Statistical analysis was performed by linear mixed models (LMM) with a randomized block design (RBD). Significant differences were assessed using the Tukey’s HSD test (p< 0.01, panel B). Asterisk indicates significant differences between the Tbx3 KO ES cell line and the parental ES cell line and different letters indicate significant differences among conditions (p< 0.05).

Standard ES cell culture conditions require LIF, which is sufficient to maintain pluripotency [47–49], and may include the ‘2i’ inhibitors set, CHIR 99021 (CHIR) and PD 0325901 (PD) that inhibit GSK3 and MEK, respectively [50], promoting together the ground state of pluripotency [51]. As mentioned above, Nanog expression is induced upon the activation of PI3K/Akt signaling pathway by LIF and the subsequent phosphorylation and inactivation of GSK3-β [10], making GSK3-β a major candidate to mediate the effect observed. Up to this point, we have demonstrated that Akt1 SUMOylatability is required for Nanog induction in ES cells routinely cultured with LIF and the 2i inhibitors set. From these observations, we thus speculated that neither GSK3-*β* nor MEK downstream pathways are essential for this regulation because they were already inhibited in our assays. In order to confirm this hypothesis, we evaluated the effect by dissecting the 2i cocktail and culturing the cells with LIF plus either CHIR or PD and with LIF alone. In all these three conditions, wild type Akt1 induced the reporter whereas 2KR Akt mutant had no effect in the studied conditions (LIF+CHIR, LIF+PD and LIF alone; Fig 3A). These results demonstrate that Akt1 SUMOylatability is required to enhance the Nanog reporter regardless of the presence of these inhibitors, revealing that neither GSK3-β nor MEK pathway are essential for this effect.

As abovementioned, Tbx3 is a key TF involved in ES cells pluripotency proposed to induce Nanog gene transcription and to be regulated by the PI3K/Akt pathway [11, 46]. Interestingly, this TF is not detected in MEFs, including the NIH/3T3 cell line [52], in which we have found that the Akt1 effect on the Nanog reporter is completely different (S2 Fig). This evidence defined this TF as a strong candidate to mediate the effect. To study if Tbx3 is essential for this effect we generated a Tbx3 knockout ES cell line by CRISPR-Cas9 technology (S4A and S4B Fig). Since Tbx3 binds to the Nanog promoter region included in the reporter used (S4C Fig), we studied the effect of Akt variants on the Nanog reporter in the KO ES cell line. As shown in Fig 3B, the basal activity of the reporter was lower in the Tbx3 KO ES cell line in comparison to the wild type parental ES cell line, as expected from the inductor activity of this TF on Nanog expression [11], thus evidencing that this reporter is responsive to Tbx3. Unexpectedly, Fig 3C shows that the absence of Tbx3 did not modify the effect of the Akt variants on the Nanog reporter assay, showing an induction by wt Akt1 and no effect with the 2KR mutant. These results absolutely exclude this candidate TF as an essential mediator of the studied effect.

In summary, the disruption of none of these two strong candidates produced loss of Nanog induction by SUMOylatable Akt, demonstrating that GSK3-β and Tbx3 are non-essential players for this effect. We might be facing a complex mechanism involving crosstalk among non-canonical pathways.

### SUMOylation susceptibility does not impact on Akt1 subcellular distribution

**Fig 4:**
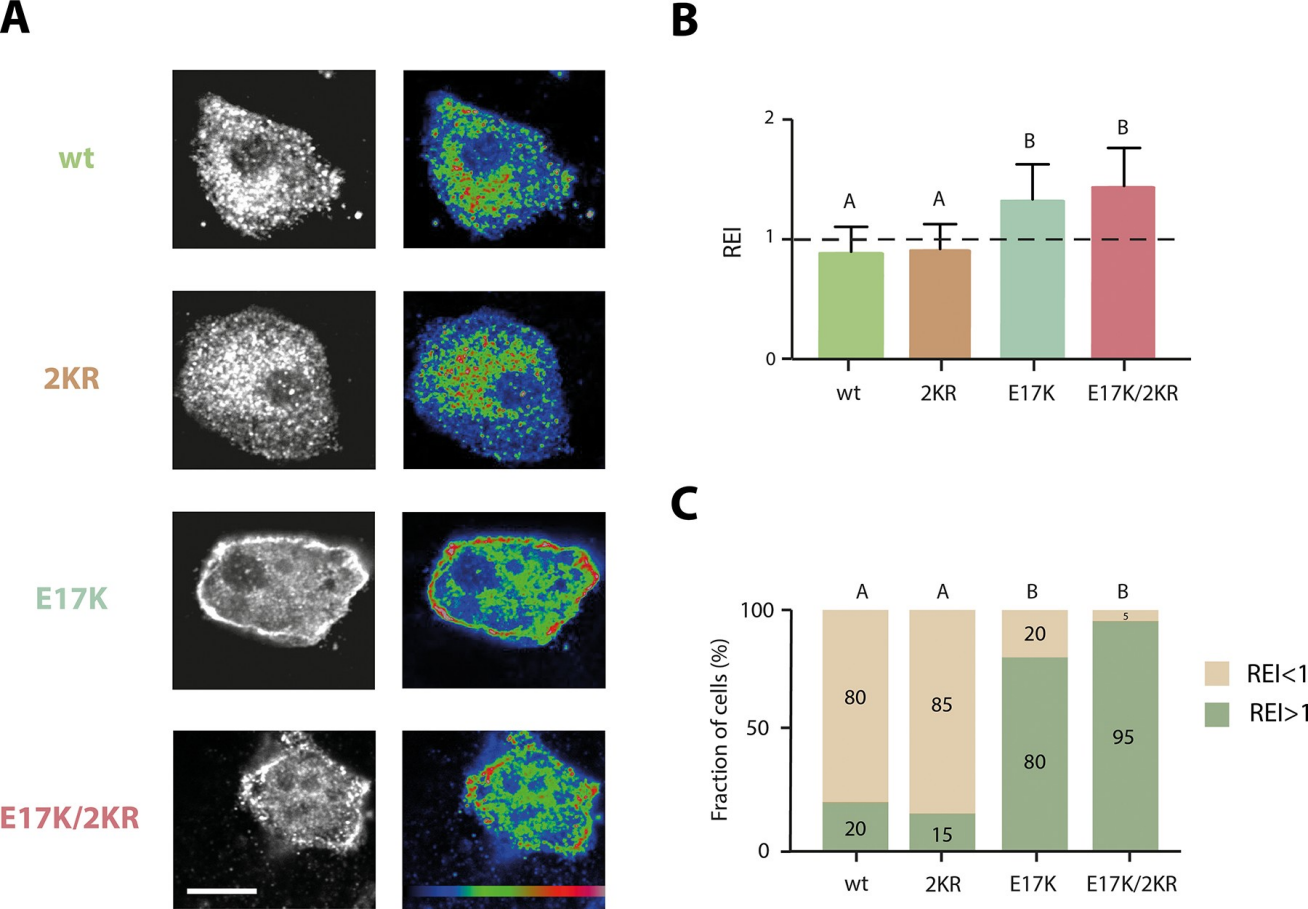
SUMOylation capability does not affect Akt1 subcellular localization. ES cells were transfected with an expression vector encoding for either wt Akt1, the Akt1 mutants 2KR, E17K or E17K/2KR. After transfection, cells were maintained in standard ES cell medium for 72 h and then fixed for immunofluorescence analysis. Transfected cells were identified by HA positive signal. (A) Representative confocal microscopy images of HA immunostaining of cells transfected with the indicated Akt1 variant. Grayscale and pseudocolor (blue to red) images of representative cells. Scale bars represent 5 μm. (B) The fluorescence intensity was quantified within a 250 nm-wide region at the cell borders and expressed relative to the mean cell intensity (relative edge intensity, REI). Bars represent mean REI ± SEM (n = 20 cells). Different letters indicate significant differences among transfectants evaluated using two-way ANOVA and Tukey’s HSD test for comparisons (p<0.0001). (C) Fraction of cells with REI values higher and lower than 1 in each condition. Different letters indicate significant differences among transfectants, evaluated using the Marascuilo’s test (p<0.05).

Finally, after discarding the major candidates for mediators of the observed effect, we focused on the subcellular distribution of Akt1 and its SUMOylatability-dependance. It is known that different subcellular distributions could define a different set of interacting proteins, thus we speculated that exploring this feature of the different Akt variants could provide a clue of the intermediate involved in this effect. For this purpose, we studied the subcellular localization of the different Akt1 variants by confocal microscopy of transfected cells. Fig 4A shows that wt Akt1 and the 2KR mutant display a qualitatively similar, relatively homogeneous distribution within the cell cytoplasm. On the other hand, the E17K mutant seems to preferentially localize at the cell boundaries, suggesting its association to the cell membrane in agreement with previous reports [42]. Remarkably, its SUMOylation-diminished counterpart, E17K/2KR, showed a similar localization, keeping the E17K phenotype. Furthermore, the quantitative analysis of the distribution of Akt variants confirmed the homogeneous distribution of wt and 2KR variants and the preferential localization at the cell boundaries of the E17K and E17K/2KR mutants (Fig 4B and 4C). These results demonstrate that Akt1 localization does not depend on its SUMOylatability, at least in this cellular context of mouse ES cells, suggesting that the different modulation of Nanog expression observed with the Akt variants is not associated to their differential subcellular distribution.

## Discussion

SUMO-conjugation modifies the activity of a wide variety of proteins with consequence in cancer and neural diseases [53, 54], and in diverse biological processes like senescence, DNA damage, replication and gene regulation at different levels [55]. In addition, SUMO modification competes with ubiquitination occurring at Lys residues in some proteins, and also cooperates with this last PTM affecting protein stability. Particularly, SUMO-conjugation of multiple TFs, including Oct4 [56] and Sox2 [57], regulates their stability, subcellular localization, DNA binding and activation capabilities [58].

Similar to other PTMs that influence Akt activity [13], SUMO-conjugation also plays a relevant role in the regulation of this kinase with consequences in multiple cellular functions such as proliferation, migration and tumorigenesis [19, 20]. Relevantly, the effect of Akt SUMOylation in stem cells was not previously explored.

In this work, we report that the SUMO-conjugation susceptibility of Akt in ES cells impacts on the capability of Akt to induce the expression of the pluripotency TF Nanog. Specifically, we found that SUMOylation-susceptible versions of Akt induced the activity of the Nanog promoter, evidenced by a reporter, and increased the levels of the endogenous Nanog protein, whereas the corresponding SUMOylatability-diminished mutants had no effect. Additionally, the lack of induction of the Nanog reporter by the Akt1 mutant that preserves K276 but has altered its SUMOylation consensus sequence, reinforces the evidence that this PTM, and not the lysine itself, is required for this effect. Finally, the abrogation of the induction exerted by the SUMOylatable Akt1 variants through co-transfection of a dominant-negative of Ubc9 reveals the role of Akt SUMOylation on Nanog promoter activity in ES cells.

Interestingly, despite the highly common oncogenic and hyperSUMOylated E17K mutant [42, 59, 60] produced a greater induction of the reporter construct than the wt version, the induction of the endogenous Nanog protein produced by both SUMOylatable versions was similar. These results suggest that an unknown regulatory mechanism may impede the overexpression of Nanog in pluripotent stem cells. In agreement, we observed that different levels of the SUMOylatable Akt variants do not produce a different increase in Nanog protein levels (Fig 2A, arrows). Noteworthy, multiple reports associate ectopic expression or anomalous activity of Nanog with oncogenic transformation [61–64] and thus this specific alteration involving Nanog induction could be also a mechanism related to the to the oncogenic properties of Akt E17K mutant.

Overall, the induction exerted on Nanog by the two SUMOylatable Akt1 variants, wt and E17K, the absence of the effect with the SUMOylatability-diminished mutants, and the interference of the induction exerted by the dominant negative mutant of the SUMO conjugase enzyme, altogether demonstrate the requirement of this Akt PTM for the regulation of the expression of the central pluripotency TF Nanog in ES cells.

Noteworthy, this effect seems to be specific to ES cells, or at least not to be part of a generalized cellular mechanism, since the response of the Nanog reporter to Akt variants was different in another cell context. We have found that all Akt1 variants repressed Nanog reporter in NIH/3T3 MEFs, a terminally differentiated cell type that does not express Nanog and which we have previously used as a heterologous non-pluripotent system in previous works [23–25]. Particularly, besides the lack of expression of the pluripotency transcription factors present in ES cells, they are also derived from mice, so they share a significant genetic background. Remarkably, the induction exerted by the SUMOylatable Akt1 variants on Nanog promoter in ES cells but not in MEFs, infers the existence of one or more essential mediators of this effect within ES cells’ context exclusively.

As mentioned above, SUMO-conjugation can modify not only the activity but the protein-protein interactions and even the proteins’ subcellular localization [16, 53, 55]. We assumed that the effect of this Akt1 PTM on Nanog expression should be mediated by at least a downstream target of this kinase which ultimately impacts on Nanog expression. To explore the possible mechanism underlying Nanog gene regulation by Akt SUMOylatability, we focused on the two main reported candidates for mediators involved in Nanog induction by Akt: GSK3-*β*, an ubiquitous kinase crucial for ES cell pluripotency maintenance [10], and Tbx3, a key pluripotency TF present in ES cells [11, 46] but not in MEFs [52]. Unexpectedly, disruption of none these factors and consequently, their downstream targets, impeded the reporter induction exerted by Akt, evidencing that they are completely dispensable for this effect. We would have expected that at least one of them was an essential mediator of this effect.

On the other hand, since it is known that subcellular localization could have an impact on the protein-target interactions, and thus protein function, we speculated that exploring the distribution of the different Akt1 variants could provide a clue of the mediator involved in this effect. The lack of a parallelism found between the distribution of the Akt variants and their effect on Nanog promoter led us to conclude that the mechanism involved is not associated with a specific Akt subcellular compartmentalization. Additionally, the obtained results strongly suggest that SUMOylation does not affect Akt1 localization in ES cells, since wt and 2KR Akt1 variants presented a similar homogeneous localization. On the other hand, the E17K mutant concentrates at the cell boundaries, in agreement with the previously reported association of this mutant with the plasma membrane [42]. Notably, its counterpart with diminished SUMOylation capability, E17K/2KR, displayed a similar distribution suggesting that SUMOylation does not affect E17K association to the membrane. These results also suggest that this PTM is a novel requirement for the hyperactivity of the E17K Akt1 mutant, since membrane localization of Akt is not sufficient to induce Nanog when its SUMO-conjugation is impaired.

We cannot rule out that, besides the effect found on Nanog reporter, SUMOylated Akt could also impact on Nanog protein stability adding an extra level of complexity on gene expression control. In this direction, there are reports of Akt phosphorylation regulating the stability of multiple proteins [65–68], emphasizing the relevance of this kinase in diverse cellular processes such as cell differentiation, unfolded protein response, oncogenesis and telomere protection. Interestingly, Nanog stability is known to be regulated by phosphorylation in ES cells [69–71] and cancer [72]. Additional studies are required to elucidate if Akt SUMOylation also influences Nanog stability.

Finally, how Akt SUMOylation is modulated in stem cells rises as another relevant question. Multiple proteins such as PIAS proteins and proteases from the SNEP family modulate either SUMO-conjugation or deconjugation to Akt, respectively, in different contexts [20]. Recently, the SUMO-activating enzyme SAE1 was found to promote human glioma progression by enhancing Akt SUMOylation-mediated signaling and propose targeting Akt SUMOylation as a promising therapeutic strategy [73]. However, further research is necessary to establish the relevance of these regulatory pathways in pluripotent stem cells in relation with Akt. Moreover, it would be of great interest to evaluate whether this Akt PTM is involved in the regulation of other relevant genes in stem cells such as other pluripotency TFs, besides Nanog. This analysis could provide information on putative unknown regulators and mediators of Akt SUMOylation in stem cells.

## Conclusion

In conclusion, we demonstrated that both the Akt SUMOylatability and a functional SUMO conjugase activity are required for the induction of Nanog expression in ES cells, highlighting the impact of this PTM in Akt function. This induction does not take place in a non-pluripotent heterologous system, strongly suggesting a context-dependent effect. Remarkably, GSK3-β and Tbx3 are non-essential players in this regulation. Furthermore, we found that Akt subcellular distribution does not depend on its SUMOylatability and that this feature is not associated with the effect observed. Further research is required to completely elucidate the players behind Nanog regulation by Akt. We speculate that we might be facing a highly complex mechanism probably involving crosstalk among non-canonical pathways. Unraveling the mechanisms that control key pluripotency TFs would enrich our understanding of stem cells’ fundamental properties.

## Supporting information

S1 Fig Akt1 variants were equally transfected. ES cells were transfected with an expression vector encoding for either wt Akt1, the Akt1 mutants E17K, 2KR, E17K/2KR or the empty vector (control). After transfection, cells were maintained in standard ES cell medium for 72 h and then fixed for immunofluorescence (A) or lysed for Western blot analysis (B). Akt variants were visualized with an antibody against HA tag. The last column shows a magnified view corresponding to the region of the yellow rectangle in each case. Scale bars: 100 μm for both columns. GAPDH was revealed as loading control. A cropped region of the whole blot is shown. Grouped lanes are indicated by a white space. Full blots are available at S1 File.
(TIF)

S2 Fig Akt1 variants repress Nanog promoter activity in a heterologous non-pluripotent system. (A) NIH/3T3 cells were transfected with Nanog5P reporter along with an expression vector encoding for either wt Akt1, the Akt1 mutants 2KR, E17K, E17K/2KR or the empty vector (basal). Luciferase activity was measured in extracts obtained 48 h after transfection. Results were referred to the control condition (basal) and are shown as mean ± SEM of three independent experiments. Statistical analysis was performed by lineal mixed models (LMM) with a randomized block design (RBD). Significant differences were assessed using the Tukey’s HSD test. Different letters indicate significant differences among cellular conditions (p< 0.05). (B) Omics data analysis of Nanog expression in MEF and ES cells. Data analysis was performed in Stemformatics (https://www.stemformatics.org) data-mining platform from fully publicly available data. Upper panel shows Nanog gene expression from RNA-seq (transcript) and LC-MS (protein) and lower panel shows epigenetic marks in Nanog promoter region from Histone CHIP-seq (H3K4me3, associated to active promoters and H3K27me3, associated to repressive marks) and genome-wide methylomic profiling experiments (associated to repressed genes). Full meta-data of analyzed datasets is available at S3 Table.
(TIF)

S3 Fig Experimental design and possible outputs. Cartoon representing the expected results of the study to explore candidate factors to mediate SUMOylatable Akt induction of Nanog reporter. The Akt1 effect was evaluated by the luciferase assay in different conditions. (i) Results obtained in control condition without inhibition. (ii, iii) Results expected for Akt1 effect evaluated in conditions of chemical inhibition, downregulation or knockout of the presumed mediators. If the factor studied is crucial for the effect, we expect no induction by wt Akt1, resulting in luciferase activity similar to that of the basal condition (ii). On the contrary, if the factor evaluated is not involved, luciferase activity should be induced by wt Akt1 (iii). In all cases Akt1 2KR mutant is evaluated as a negative control of reporter induction since this mutant has no effect on the reporter.
(TIF)

S4 Fig Generation of Txb3 knockout (KO) ES cell line and Tbx3 binding on Nanog promoter region used. (A) Generation of Txb3 knockout (KO) ES cell line: Diagram of the Tbx3 gene (violet), mRNA (green) and the CRISPR-guided cleavage sites (red). CRISPR sgRNAs sgTBX3m E1 and E2 targeted the first and second coding exons, respectively [30]. The illustration was prepared using SnapGene Viewer (GSL Biotech; available at snapgene.com). (B) Left panel shows representative images of TBX3 immunostaining for wt and Tbx3-KO ES cell lines. Scale bar: 10 μm. Right panel shows Western blot demonstrating the absence of Tbx3 protein in Tbx3-KO ES cell line. Full blots are available at S1 File. (C) Visualization of representative enrichment profile (reads per million) of the Tbx3 TF in the 2.5 kpb region of the Nanog genomic locus included in Nanog5P reporter. The results shown correspond to the analysis of public ChIP-seq data from experiments performed in ES cells (Chip Atlas database: http://chip-atlas.org) [34]. Data was visualized using the Integrative Genomics Viewer (IGV) software [35]. Full meta-data of analyzed datasets is available at S3 Table.
(TIF)

S1 Table Antibodies used. List of the antibodies used in this work.
(XLSX)

S2 Table CRISPR sgRNA sequences. List of the sgRNA sequences used for the generation of the Tbx3 CRISPR-guided knockout ES cell line used in this work.
(XLSX)

S3 Table Omics datasets analyzed in this work. Publicly available datasets used in this work.
(XLSX)

S1 File Full blots. Uncropped blots of the western blot figures shown in this work.
(PDF)

## References

[1] Evans MJ, Kaufman MH. Establishment in culture of pluripotential cells from mouse embryos. Nature [Internet]. 1981;292(5819):154–6. Available from: http://www.ncbi.nlm.nih.gov/entrez/query.fcgi?cmd=Retrieve&db=PubMed&dopt=Citation&list_uids=7242681 doi: 10.1038/292154a0 7242681

[2] Martin GR. Isolation of a pluripotent cell line from early mouse embryos cultured in medium conditioned by teratocarcinoma stem cells. Proc Natl Acad Sci U S A. 1981;78(12):7634–8. doi: 10.1073/pnas.78.12.7634 6950406 PMC349323

[3] Williams RL, Hilton DJ, Pease S, Willson TA, Stewart CL, Gearing DP, et al. Myeloid leukaemia inhibitory factor maintains the developmental potential of embryonic stem cells. Nature. 1988 Dec;336(6200):684–7. doi: 10.1038/336684a0 3143916

[4] Ohtsuka S, Nakai-Futatsugi Y, Niwa H. LIF signal in mouse embryonic stem cells. JAK-STAT [Internet]. 2015 Apr 3 [cited 2017 Aug 3];4(2):1–9. Available from: http://www.ncbi.nlm.nih.gov/pubmed/27127728 doi: 10.1080/21623996.2015.1086520 27127728 PMC4802755

[5] Loh Y-H, Wu Q, Chew J-L, Vega VB, Zhang W, Chen X, et al. The Oct4 and Nanog transcription network regulates pluripotency in mouse embryonic stem cells. Nat Genet [Internet]. 2006 Apr 5 [cited 2018 Dec 6];38(4):431–40. Available from: http://www.nature.com/articles/ng1760 doi: 10.1038/ng1760 16518401

[6] Zhou Q, Chipperfield H, Melton DA, Wong WH. A gene regulatory network in mouse embryonic stem cells. Proc Natl Acad Sci U S A. 2007 Oct;104(42):16438–43. doi: 10.1073/pnas.0701014104 17940043 PMC2034259

[7] Mitsui K, Tokuzawa Y, Itoh H, Segawa K, Murakami M, Takahashi K, et al. The homeoprotein Nanog is required for maintenance of pluripotency in mouse epiblast and ES cells. Cell [Internet]. 2003;113(5):631–42. Available from: http://www.ncbi.nlm.nih.gov/entrez/query.fcgi?cmd=Retrieve&db=PubMed&dopt=Citation&list_uids=12787504 doi: 10.1016/s0092-8674(03)00393-3 12787504

[8] Chambers I, Colby D, Robertson M, Nichols J, Lee S, Tweedie S, et al. Functional expression cloning of Nanog, a pluripotency sustaining factor in embryonic stem cells. Cell [Internet]. 2003 May;113(5):643–55. Available from: http://www.ncbi.nlm.nih.gov/entrez/query.fcgi?cmd=Retrieve&db=PubMed&dopt=Citation&list_uids=12787505 doi: 10.1016/s0092-8674(03)00392-1 12787505

[9] Saunders A, Faiola F, Wang J. Concise review: pursuing self-renewal and pluripotency with the stem cell factor Nanog. Stem Cells [Internet]. 2013 Jul [cited 2019 Feb 1];31(7):1227–36. Available from: http://www.ncbi.nlm.nih.gov/pubmed/23653415 doi: 10.1002/stem.1384 23653415 PMC3706551

[10] Storm MP, Bone HK, Beck CG, Bourillot P-Y, Schreiber V, Damiano T, et al. Regulation of Nanog Expression by Phosphoinositide 3-Kinase-dependent Signaling in Murine Embryonic Stem Cells. J Biol Chem [Internet]. 2007 Mar 2 [cited 2018 Oct 24];282(9):6265–73. Available from: http://www.ncbi.nlm.nih.gov/pubmed/17204467 doi: 10.1074/jbc.M610906200 17204467

[11] Niwa H, Ogawa K, Shimosato D, Adachi K. A parallel circuit of LIF signalling pathways maintains pluripotency of mouse ES cells. Nature [Internet]. 2009;460(7251):118–22. Available from: 10.1038/nature08113 19571885

[12] Hers I, Vincent EE, Tavaré JM. Akt signalling in health and disease. Vol. 23, Cellular Signalling. Cell Signal; 2011. p. 1515–27. doi: 10.1016/j.cellsig.2011.05.004 21620960

[13] Risso G, Blaustein M, Pozzi B, Mammi P, Srebrow A. Akt/PKB: one kinase, many modifications. Biochem J [Internet]. 2015 Jun 1 [cited 2017 Jun 7];468(2):203–14. Available from: http://www.ncbi.nlm.nih.gov/pubmed/25997832 doi: 10.1042/BJ20150041 25997832

[14] Hanada M, Feng J, Hemmings BA. Structure, regulation and function of PKB/AKT—a major therapeutic target. Biochim Biophys Acta—Proteins Proteomics. 2004 Mar;1697(1–2):3–16. 10.1016/j.bbapap.2003.11.009 15023346

[15] Watanabe S, Umehara H, Murayama K, Okabe M, Kimura T, Nakano T. Activation of Akt signaling is sufficient to maintain pluripotency in mouse and primate embryonic stem cells. Oncogene. 2006;25(19):2697–707. doi: 10.1038/sj.onc.1209307 16407845

[16] Flotho A, Melchior F. Sumoylation: A Regulatory Protein Modification in Health and Disease. Annu Rev Biochem. 2013 Jun;82(1):357–85. doi: 10.1146/annurev-biochem-061909-093311 23746258

[17] Melchior F. SUMO—Nonclassical ubiquitin [Internet]. Vol. 16, Annual Review of Cell and Developmental Biology. Annual Reviews 4139 El Camino Way, P.O. Box 10139, Palo Alto, CA 94303–0139, USA; 2000 [cited 2021 May 7]. p. 591–626. Available from: https://www.annualreviews.org/doi/abs/10.1146/annurev.cellbio.16.1.591 10.1146/annurev.cellbio.16.1.591 11031248

[18] Pichler A, Fatouros C, Lee H, Eisenhardt N. SUMO conjugation–a mechanistic view. Biomol Concepts. 2017 Jan;8(1):13–36. doi: 10.1515/bmc-2016-0030 28284030

[19] Risso G, Pelisch F, Pozzi B, Mammi P, Blaustein M, Colman-Lerner A, et al. Modification of Akt by SUMO conjugation regulates alternative splicing and cell cycle. Cell Cycle [Internet]. 2013 Oct 1 [cited 2016 Aug 19];12(19):3165–74. Available from: http://www.ncbi.nlm.nih.gov/pubmed/24013425 doi: 10.4161/cc.26183 24013425 PMC3865012

[20] Li R, Wei J, Jiang C, Liu D, Deng L, Zhang K, et al. Akt SUMOylation Regulates Cell Proliferation and Tumorigenesis. Cancer Res [Internet]. 2013 Sep 15 [cited 2018 Jul 10];73(18):5742–53. Available from: http://www.ncbi.nlm.nih.gov/pubmed/23884910 doi: 10.1158/0008-5472.CAN-13-0538 23884910

[21] De La Cruz-Herrera CF, Campagna M, Lang V, Del Carmen González-Santamaría J, Marcos-Villar L, Rodríguez MS, et al. SUMOylation regulates AKT1 activity. Oncogene [Internet]. 2015;34(11):1442–50. Available from: http://www.ncbi.nlm.nih.gov/pubmed/24704831 doi: 10.1038/onc.2014.48 24704831

[22] Lin CH, Liu SY, Lee EHY. SUMO modification of Akt regulates global SUMOylation and substrate SUMOylation specificity through Akt phosphorylation of Ubc9 and SUMO1. Oncogene [Internet]. 2016 Feb 4 [cited 2016 Sep 7];35(5):595–607. Available from: http://www.ncbi.nlm.nih.gov/pubmed/25867063 doi: 10.1038/onc.2015.115 25867063

[23] Solari C, Echegaray CV, Cosentino MS, Petrone MV, Waisman A, Luzzani C, et al. Manganese superoxide dismutase gene expression is induced by Nanog and Oct4, essential pluripotent stem cells’ transcription factors. PLoS One [Internet]. 2015 Jan [cited 2015 Dec 13];10(12):e0144336. Available from: http://www.pubmedcentral.nih.gov/articlerender.fcgi?artid=4671669&tool=pmcentrez&rendertype=abstract doi: 10.1371/journal.pone.0144336 26642061 PMC4671669

[24] Solari C, Petrone MV, Vazquez Echegaray C, Cosentino MS, Waisman A, Francia M, et al. Superoxide dismutase 1 expression is modulated by the core pluripotency transcription factors Oct4, Sox2 and Nanog in embryonic stem cells. Mech Dev [Internet]. 2018 Jun 19 [cited 2018 Jul 30];154:116–21. Available from: http://www.ncbi.nlm.nih.gov/pubmed/29933066 doi: 10.1016/j.mod.2018.06.004 29933066

[25] Solari C, Echegaray CV, Luzzani C, Cosentino MS, Waisman A, Petrone MV, et al. Protein arginine Methyltransferase 8 gene is expressed in pluripotent stem cells and its expression is modulated by the transcription factor Sox2. Biochem Biophys Res Commun. 2016;473(1). doi: 10.1016/j.bbrc.2016.03.077 27012206

[26] Gu P, LeMenuet D, Chung AC-K, Mancini M, Wheeler DA, Cooney AJ. Orphan Nuclear Receptor GCNF Is Required for the Repression of Pluripotency Genes during Retinoic Acid-Induced Embryonic Stem Cell Differentiation. Mol Cell Biol. 2005;25(19):8507–19. doi: 10.1128/MCB.25.19.8507-8519.2005 16166633 PMC1265758

[27] Carbia-Nagashima A, Gerez J, Perez-Castro C, Paez-Pereda M, Silberstein S, Stalla GK, et al. RSUME, a Small RWD-Containing Protein, Enhances SUMO Conjugation and Stabilizes HIF-1α during Hypoxia. Cell. 2007 Oct 19;131(2):309–23. doi: 10.1016/j.cell.2007.07.044 17956732

[28] Cosentino MS, Oses C, Vázquez Echegaray C, Solari C, Waisman A, Álvarez Y, et al. Kat6b Modulates Oct4 and Nanog Binding to Chromatin in Embryonic Stem Cells and Is Required for Efficient Neural Differentiation. J Mol Biol [Internet]. 2019;431(6):1148–59. Available from: 10.1016/j.jmb.2019.02.012 30790630

[29] Waisman A, Vazquez Echegaray C, Solari C, Cosentino MS, Martyn I, Deglincerti A, et al. Inhibition of Cell Division and DNA Replication Impair Mouse-Naïve Pluripotency Exit. J Mol Biol. 2017;429(18):2802–15. doi: 10.1016/j.jmb.2017.06.020 28684247

[30] Oh TJ, Adhikari A, Mohamad T, Althobaiti A, Davie J. TBX3 represses TBX2 under the control of the PRC2 complex in skeletal muscle and rhabdomyosarcoma. Oncogenesis. 2019 Apr;8(4). doi: 10.1038/s41389-019-0137-z 30979887 PMC6461654

[31] Verneri P, Vazquez Echegaray C, Oses C, Stortz M, Guberman A, Levi V, et al. Dynamical reorganization of the pluripotency transcription factors Oct4 and Sox2 during early differentiation of embryonic stem cells. Sci Rep. 2020 Dec 1;10(1):1–12. doi: 10.1038/s41598-019-56847-4 32251342 PMC7089971

[32] Wells CA, Mosbergen R, Korn O, Choi J, Seidenman N, Matigian NA, et al. Stemformatics: Visualisation and sharing of stem cell gene expression. Stem Cell Res [Internet]. 2013 May [cited 2021 May 14];10(3):387–95. Available from: https://pubmed.ncbi.nlm.nih.gov/23466562/ doi: 10.1016/j.scr.2012.12.003 23466562

[33] Choi J, Pacheco CM, Mosbergen R, Korn O, Chen T, Nagpal I, et al. Stemformatics: Visualize and download curated stem cell data. Nucleic Acids Res [Internet]. 2019 Jan 8 [cited 2021 May 14];47(D1):D841–6. Available from: https://www. doi: 10.1093/nar/gky1064 30407577 PMC6323943

[34] Oki S, Ohta T, Shioi G, Hatanaka H, Ogasawara O, Okuda Y, et al. ChIP-Atlas: a data-mining suite powered by full integration of public ChIP-seq data. EMBO Rep. 2018 Dec;19(12). doi: 10.15252/embr.201846255 30413482 PMC6280645

[35] Robinson JT, Thorvaldsdóttir H, Winckler W, Guttman M, Lander ES, Getz G, et al. Integrative genomics viewer. Vol. 29, Nature Biotechnology. NIH Public Access; 2011. p. 24–6. doi: 10.1038/nbt.1754 21221095 PMC3346182

[36] Di Rienzo JA, Guzman AW, Casanoves F. A multiple-comparisons method based on the distribution of the root node distance of a binary tree. J Agric Biol Environ Stat. 2002;7(2):129–42.

[37] di Rienzo JA, Casanoves F, Balzarini M, Gonzalez L, Tablada M, Robledo C. Infostat—Sofware estadístico. Universidad Nacional de Córdoba, Argentina. Universidad Nacional de Córdoba, Argentina. 2013.

[38] Marascuilo LA. Large-sample multiple comparisons. Psychol Bull [Internet]. 1966 May [cited 2020 Jan 13];65(5):280–90. Available from: http://www.ncbi.nlm.nih.gov/pubmed/5325892 doi: 10.1037/h0023189 5325892

[39] Dinh TTH, Iseki H, Mizuno S, Iijima-Mizuno S, Tanimoto Y, Daitoku Y, et al. Cables2 is a novel Smad2-regulatory factor essential for early embryonic development in mice. bioRxiv [Internet]. 2019 Aug 22 [cited 2020 Apr 24];14. Available from: 10.1101/744128

[40] Po A, Ferretti E, Miele E, De Smaele E, Paganelli A, Canettieri G, et al. Hedgehog controls neural stem cells through p53-independent regulation of Nanog. EMBO J [Internet]. 2010 Aug 4 [cited 2020 Apr 24];29(15):2646–58. Available from: http://emboj.embopress.org/cgi/doi/10.1038/emboj.2010.131 20581804 10.1038/emboj.2010.131 PMC2928686

[41] Wang Q, Xu X, Li J, Liu J, Gu H, Zhang R, et al. Lithium, an anti-psychotic drug, greatly enhances the generation of induced pluripotent stem cells. Cell Res. 2011 Oct 5;21(10):1424–35. doi: 10.1038/cr.2011.108 21727907 PMC3193456

[42] Carpten JD, Faber AL, Horn C, Donoho GP, Briggs SL, Robbins CM, et al. A transforming mutation in the pleckstrin homology domain of AKT1 in cancer. Nature [Internet]. 2007 Jul 26 [cited 2016 Apr 6];448(7152):439–44. Available from: http://www.nature.com/articles/nature05933 doi: 10.1038/nature05933 17611497

[43] Benevento M, Tonge PD, Puri MC, Hussein SMI, Cloonan N, Wood DL, et al. Proteome adaptation in cell reprogramming proceeds via distinct transcriptional networks. Nat Commun [Internet]. 2014 Dec 10 [cited 2021 May 14];5(1):1–11. Available from: www.nature.com/naturecommunications doi: 10.1038/ncomms6613 25494451

[44] Hussein SMI, Puri MC, Tonge PD, Benevento M, Corso AJ, Clancy JL, et al. Genome-wide characterization of the routes to pluripotency. Nature [Internet]. 2014 Dec 11 [cited 2021 May 14];516(7530):198–206. Available from: https://www.nature.com/articles/nature14046 doi: 10.1038/nature14046 25503233

[45] Gong L, Kamitani T, Fujise K, Caskey LS, Yeh ETH. Preferential interaction of sentrin with a ubiquitin-conjugating enzyme, Ubc9. J Biol Chem [Internet]. 1997 Nov 7 [cited 2021 May 10];272(45):28198–201. Available from: https://pubmed.ncbi.nlm.nih.gov/9353268/ doi: 10.1074/jbc.272.45.28198 9353268

[46] Ivanova N, Dobrin R, Lu R, Kotenko I, Levorse J, DeCoste C, et al. Dissecting self-renewal in stem cells with RNA interference. Nature [Internet]. 2006;442(7102):533–8. Available from: https://www.nature.com/articles/nature04915#supplementary-information doi: 10.1038/nature04915 16767105

[47] Smith AG, Heath JK, Donaldson DD, Wong GG, Moreau J, Stahl M, et al. Inhibition of pluripotential embryonic stem cell differentiation by purified polypeptides. Nature [Internet]. 1988;336(6200):688–90. Available from: http://www.ncbi.nlm.nih.gov/entrez/query.fcgi?cmd=Retrieve&db=PubMed&dopt=Citation&list_uids=3143917 doi: 10.1038/336688a0 3143917

[48] Niwa H, Burdon T, Chambers I, Smith A. Self-renewal of pluripotent embryonic stem cells is mediated via activation of STAT3. Genes Dev [Internet]. 1998;12(13):2048–60. Available from: http://www.ncbi.nlm.nih.gov/entrez/query.fcgi?cmd=Retrieve&db=PubMed&dopt=Citation&list_uids=9649508 doi: 10.1101/gad.12.13.2048 9649508 PMC316954

[49] Matsuda T, Nakamura T, Nakao K, Arai T, Katsuki M, Heike T, et al. STAT3 activation is sufficient to maintain an undifferentiated state of mouse embryonic stem cells. EMBO J [Internet]. 1999 Aug 2 [cited 2018 Oct 24];18(15):4261–9. Available from: http://www.ncbi.nlm.nih.gov/pubmed/10428964 doi: 10.1093/emboj/18.15.4261 10428964 PMC1171502

[50] Ying Q-L, Wray J, Nichols J, Batlle-Morera L, Doble B, Woodgett J, et al. The ground state of embryonic stem cell self-renewal. Nature. 2008;453(7194):519–23. doi: 10.1038/nature06968 18497825 PMC5328678

[51] Ying Q-L, Wray J, Nichols J, Batlle-Morera L, Doble B, Woodgett J, et al. The ground state of embryonic stem cell self-renewal. Nature. 2008;453:519–23. doi: 10.1038/nature06968 18497825 PMC5328678

[52] Lu R, Yang A, Jin Y. Dual functions of T-box 3 (Tbx3) in the control of self-renewal and extraembryonic endoderm differentiation in mouse embryonic stem cells. J Biol Chem [Internet]. 2011 Mar 11 [cited 2021 May 14];286(10):8425–36. Available from: http://www.jbc.org doi: 10.1074/jbc.M110.202150 21189255 PMC3048727

[53] Han Z-J, Feng Y-H, Gu B-H, Li Y-M, Chen H, Gu B-H, et al. The post-translational modification, SUMOylation, and cancer (Review). Int J Oncol [Internet]. 2018 Feb 22 [cited 2019 Jan 8];52(4):1081–94. Available from: http://www.spandidos-publications.com/10.3892/ijo.2018.4280 29484374 10.3892/ijo.2018.4280 PMC5843405

[54] Yang Y, He Y, Wang X, Liang Z, He G, Zhang P, et al. Protein SUMOylation modification and its associations with disease. Open Biol [Internet]. 2017 Oct 11 [cited 2019 Jan 8];7(10):170167. Available from: http://rsob.royalsocietypublishing.org/lookup/doi/10.1098/rsob.170167 29021212 10.1098/rsob.170167 PMC5666083

[55] Zhao X. SUMO-Mediated Regulation of Nuclear Functions and Signaling Processes. Mol Cell [Internet]. 2018 Aug 2 [cited 2019 Jan 8];71(3):409–18. Available from: http://www.ncbi.nlm.nih.gov/pubmed/30075142 doi: 10.1016/j.molcel.2018.07.027 30075142 PMC6095470

[56] Wei F, Schöler HR, Atchison ML. Sumoylation of Oct4 enhances its stability, DNA binding, and transactivation. J Biol Chem [Internet]. 2007 Jul 20 [cited 2019 Jan 8];282(29):21551–60. Available from: http://www.jbc.org/lookup/doi/10.1074/jbc.M611041200 17525163 10.1074/jbc.M611041200

[57] Tsuruzoe S, Ishihara K, Uchimura Y, Watanabe S, Sekita Y, Aoto T, et al. Inhibition of DNA binding of Sox2 by the SUMO conjugation. Biochem Biophys Res Commun [Internet]. 2006 Dec 29 [cited 2019 Jan 8];351(4):920–6. Available from: https://www.sciencedirect.com/science/article/pii/S0006291X06023904 doi: 10.1016/j.bbrc.2006.10.130 17097055

[58] Gill G. Post-translational modification by the small ubiquitin-related modifier SUMO has big effects on transcription factor activity. Curr Opin Genet Dev [Internet]. 2003 Apr [cited 2019 Jan 8];13(2):108–13. Available from: http://www.ncbi.nlm.nih.gov/pubmed/12672486 doi: 10.1016/s0959-437x(03)00021-2 12672486

[59] Malanga D, Belmonte S, Colelli F, Scarfò M, De Marco C, Oliveira DM, et al. AKT1E17K is oncogenic in mouse lung and cooperates with chemical carcinogens in inducing lung cancer. PLoS One. 2016;11(2). doi: 10.1371/journal.pone.0147334 26859676 PMC4747507

[60] De Marco C, Malanga D, Rinaldo N, De Vita F, Scrima M, Lovisa S, et al. Mutant AKT1-E17K is oncogenic in lung epithelial cells. Oncotarget. 2015;6(37):39634–50. doi: 10.18632/oncotarget.4022 26053093 PMC4741851

[61] de Vicente JC, Rodríguez-Santamarta T, Rodrigo JP, Allonca E, Vallina A, Singhania A, et al. The Emerging Role of NANOG as an Early Cancer Risk Biomarker in Patients with Oral Potentially Malignant Disorders. J Clin Med [Internet]. 2019 Sep 3 [cited 2020 Jan 9];8(9):1376. Available from: http://www.ncbi.nlm.nih.gov/pubmed/31484317 doi: 10.3390/jcm8091376 31484317 PMC6780631

[62] Rasti A, Mehrazma M, Madjd Z, Abolhasani M, Saeednejad Zanjani L, Asgari M. Co-expression of Cancer Stem Cell Markers OCT4 and NANOG Predicts Poor Prognosis in Renal Cell Carcinomas. Sci Rep [Internet]. 2018 Dec 6 [cited 2020 Jan 9];8(1):11739. Available from: http://www.nature.com/articles/s41598-018-30168-4 doi: 10.1038/s41598-018-30168-4 30082842 PMC6079110

[63] Luo W, Li S, Peng B, Ye Y, Deng X, Yao K. Embryonic Stem Cells Markers SOX2, OCT4 and Nanog Expression and Their Correlations with Epithelial-Mesenchymal Transition in Nasopharyngeal Carcinoma. Aziz SA, editor. PLoS One [Internet]. 2013 Feb 12 [cited 2020 Jan 9];8(2):e56324. Available from: http://dx.plos.org/10.1371/journal.pone.0056324 23424657 10.1371/journal.pone.0056324 PMC3570418

[64] Rodrigo JP, Villaronga MÁ, Menéndez ST, Hermida-Prado F, Quer M, Vilaseca I, et al. A Novel Role For Nanog As An Early Cancer Risk Marker In Patients With Laryngeal Precancerous Lesions. Sci Rep [Internet]. 2017 Dec 11 [cited 2020 Jan 9];7(1):11110. Available from: http://www.nature.com/articles/s41598-017-11709-9 doi: 10.1038/s41598-017-11709-9 28894270 PMC5594002

[65] Choi YH, Kim Y-J, Jeong HM, Jin Y-H, Yeo C-Y, Lee KY. Akt enhances Runx2 protein stability by regulating Smurf2 function during osteoblast differentiation. FEBS J [Internet]. 2014 Aug [cited 2019 Jan 22];281(16):3656–66. Available from: http://www.ncbi.nlm.nih.gov/pubmed/24961731 doi: 10.1111/febs.12887 24961731

[66] Wang Y, Zhang Y, Yi P, Dong W, Nalin AP, Zhang J, et al. The IL-15–AKT–XBP1s signaling pathway contributes to effector functions and survival in human NK cells. Nat Immunol [Internet]. 2019 Jan 10 [cited 2019 Jan 22];20(1):10–7. Available from: http://www.ncbi.nlm.nih.gov/pubmed/30538328 doi: 10.1038/s41590-018-0265-1 30538328 PMC6293989

[67] Wang VY-F, Li Y, Kim D, Zhong X, Du Q, Ghassemian M, et al. Bcl3 Phosphorylation by Akt, Erk2, and IKK Is Required for Its Transcriptional Activity. Mol Cell [Internet]. 2017 Aug 3 [cited 2019 Jan 22];67(3):484–497.e5. Available from: http://www.ncbi.nlm.nih.gov/pubmed/28689659 doi: 10.1016/j.molcel.2017.06.011 28689659 PMC6571149

[68] Méndez-Pertuz M, Martínez P, Blanco-Aparicio C, Gómez-Casero E, Belen García A, Martínez-Torrecuadrada J, et al. Modulation of telomere protection by the PI3K/AKT pathway. Nat Commun [Internet]. 2017 Dec 2 [cited 2019 Jan 22];8(1):1278. Available from: http://www.ncbi.nlm.nih.gov/pubmed/29097657 doi: 10.1038/s41467-017-01329-2 29097657 PMC5668434

[69] Kim SH, Kim MO, Cho YY, Yao K, Kim DJ, Jeong CH, et al. ERK1 phosphorylates Nanog to regulate protein stability and stem cell self-renewal. Stem Cell Res [Internet]. 2014;13(1):1–11. Available from: 10.1016/j.scr.2014.04.001 24793005

[70] Moretto-Zita M, Jin H, Shen Z, Zhao T, Briggs SP, Xu Y. Phosphorylation stabilizes Nanog by promoting its interaction with Pin1. Proc Natl Acad Sci U S A. 2010;107(30):13312–7. doi: 10.1073/pnas.1005847107 20622153 PMC2922169

[71] Ramakrishna S, Suresh B, Lim KH, Cha BH, Lee SH, Kim KS, et al. PEST motif sequence regulating human NANOG for proteasomal degradation. Stem Cells Dev. 2011;20(9):1511–9. doi: 10.1089/scd.2010.0410 21299413

[72] Zhang J, Chen M, Zhu Y, Dai X, Dang F, Ren J, et al. SPOP Promotes Nanog Destruction to Suppress Stem Cell Traits and Prostate Cancer Progression. Dev Cell. 2019;48(3):329–344.e5. doi: 10.1016/j.devcel.2018.11.035 30595538 PMC6462403

[73] Yang Y, Liang Z, Xia Z, Wang X, Ma Y, Sheng Z, et al. SAE1 promotes human glioma progression through activating AKT SUMOylation-mediated signaling pathways. Cell Commun Signal. 2019 Jul 25;17(1). doi: 10.1186/s12964-019-0392-9 31345225 PMC6659289

