# Supplementary figures and images for: SUMO conjugation susceptibility of Akt/protein kinase B affects the expression of the pluripotency transcription factor Nanog in embryonic stem cells
Source: PLoS One. 2021 Jul 9;16(7):e0254447. doi: 10.1371/journal.pone.0254447 (PMC8270172; doi:10.1371/journal.pone.0254447)

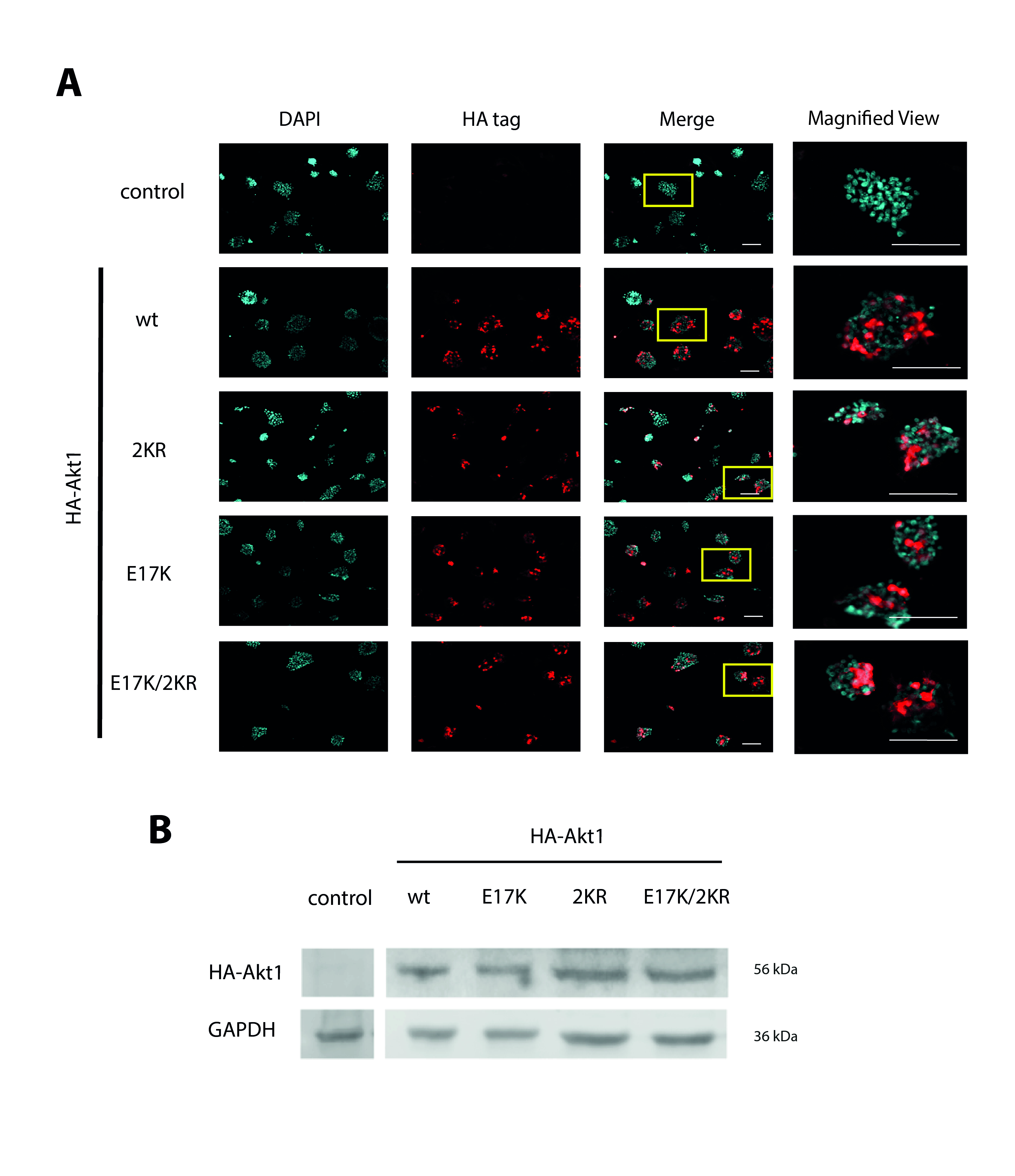

Supplement: S1 Fig — ES cells were transfected with an expression vector encoding for either wt Akt1, the Akt1 mutants E17K, 2KR, E17K/2KR or the empty vector (control). After transfection, cells were maintained in standard ES cell medium for 72 h and then fixed for immunofluorescence (A) or lysed for Western blot analysis (B). Akt variants were visualized with an antibody against HA tag. The last column shows a magnified view corresponding to the region of the yellow rectangle in each case. Scale bars: 100 μm for both columns. GAPDH was revealed as loading control. A cropped region of the whole blot is shown. Grouped lanes are indicated by a white space. Full blots are available at S1 File. (TIF) [file pone.0254447.s001.tif]

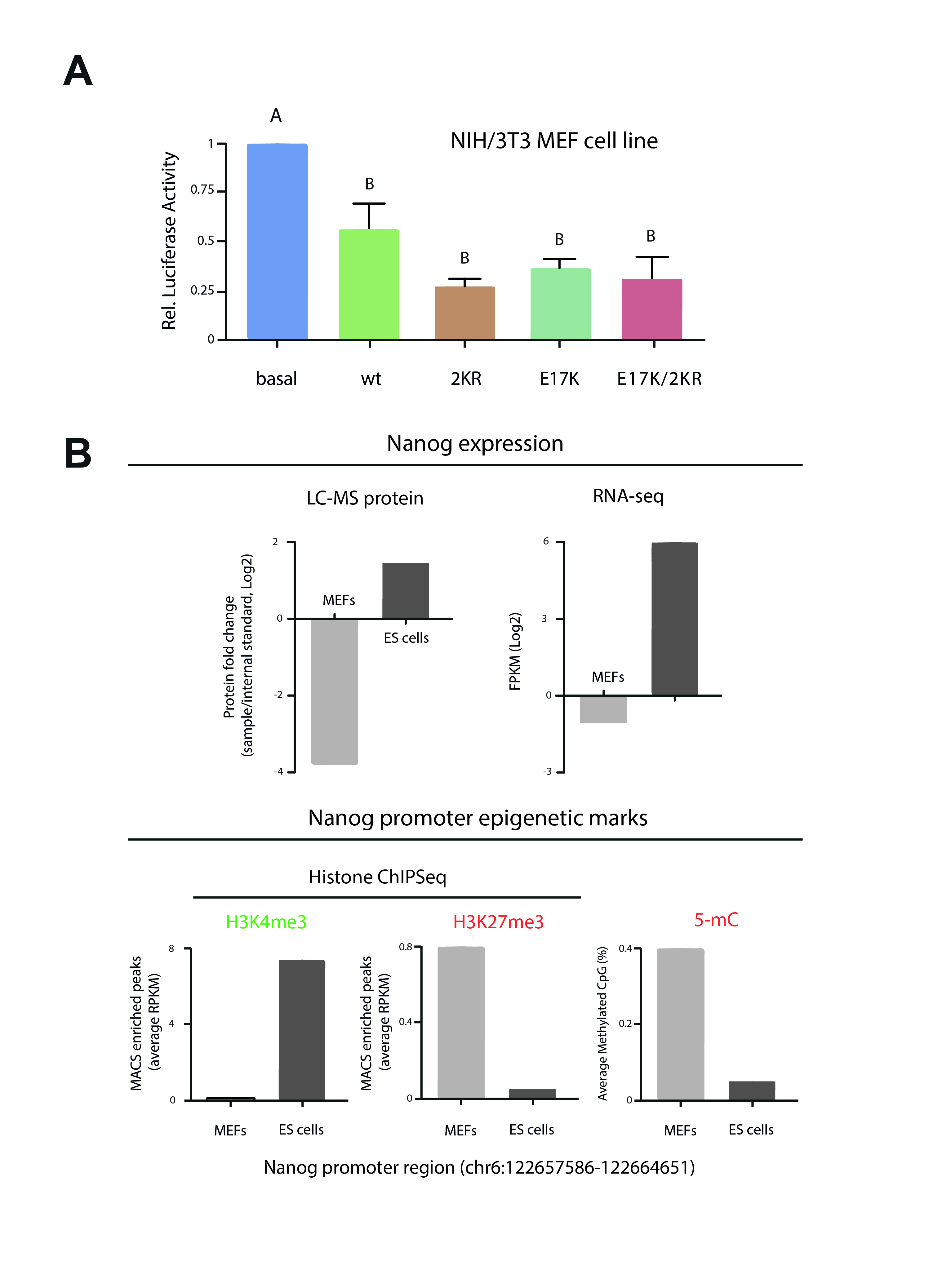

Supplement: S2 Fig — (A) NIH/3T3 cells were transfected with Nanog5P reporter along with an expression vector encoding for either wt Akt1, the Akt1 mutants 2KR, E17K, E17K/2KR or the empty vector (basal). Luciferase activity was measured in extracts obtained 48 h after transfection. Results were referred to the control condition (basal) and are shown as mean ± SEM of three independent experiments. Statistical analysis was performed by lineal mixed models (LMM) with a randomized block design (RBD). Significant differences were assessed using the Tukey’s HSD test. Different letters indicate significant differences among cellular conditions (p< 0.05). (B) Omics data analysis of Nanog expression in MEF and ES cells. Data analysis was performed in Stemformatics (https://www.stemformatics.org) data-mining platform from fully publicly available data. Upper panel shows Nanog gene expression from RNA-seq (transcript) and LC-MS (protein) and lower panel shows epigenetic marks in Nanog promoter region from Histone CHIP-seq (H3K4me3, associated to active promoters and H3K27me3, associated to repressive marks) and genome-wide methylomic profiling experiments (associated to repressed genes). Full meta-data of analyzed datasets is available at S3 Table. (TIF) [file pone.0254447.s002.tif]

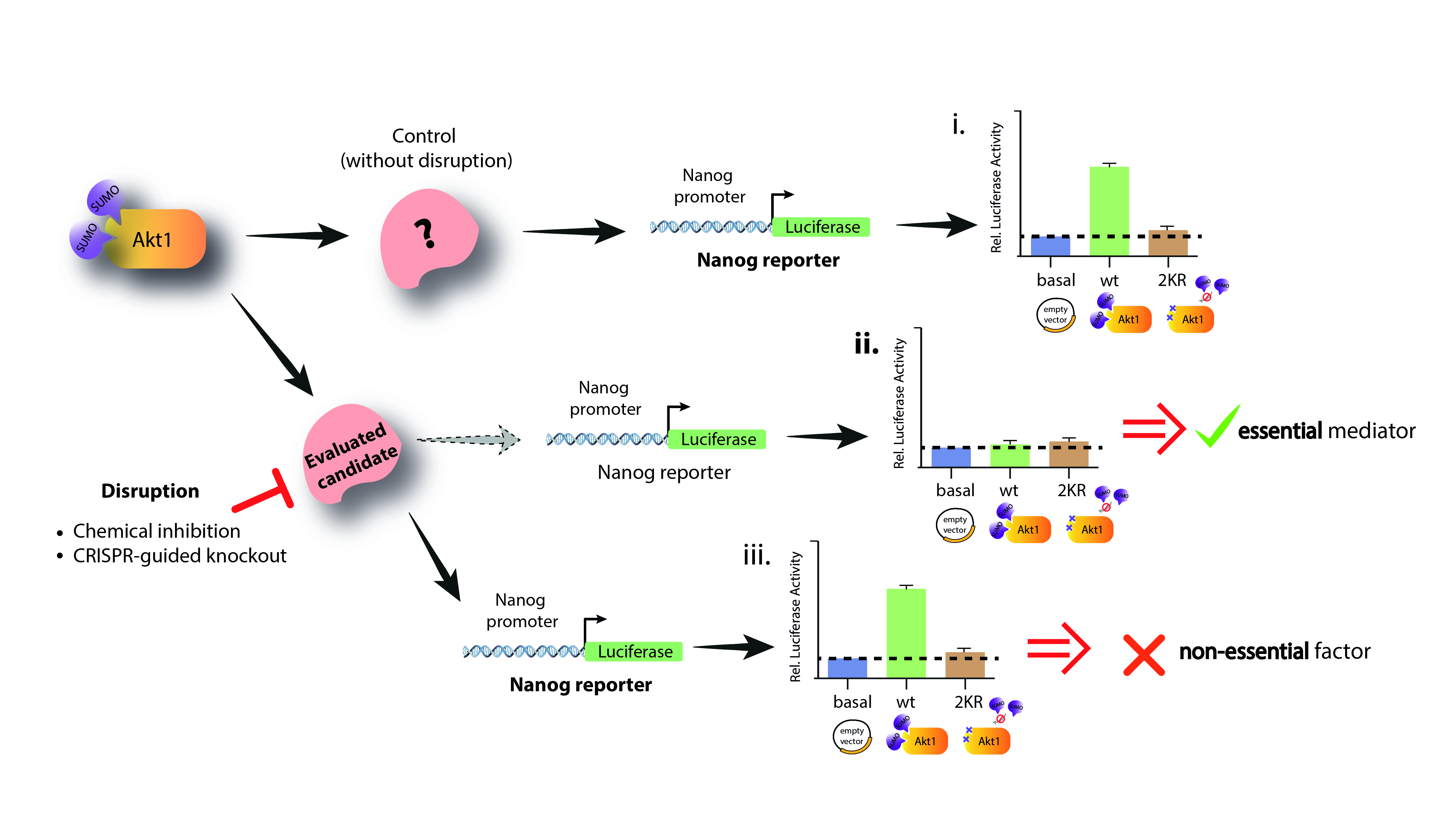

Supplement: S3 Fig — Cartoon representing the expected results of the study to explore candidate factors to mediate SUMOylatable Akt induction of Nanog reporter. The Akt1 effect was evaluated by the luciferase assay in different conditions. (i) Results obtained in control condition without inhibition. (ii, iii) Results expected for Akt1 effect evaluated in conditions of chemical inhibition, downregulation or knockout of the presumed mediators. If the factor studied is crucial for the effect, we expect no induction by wt Akt1, resulting in luciferase activity similar to that of the basal condition (ii). On the contrary, if the factor evaluated is not involved, luciferase activity should be induced by wt Akt1 (iii). In all cases Akt1 2KR mutant is evaluated as a negative control of reporter induction since this mutant has no effect on the reporter. (TIF) [file pone.0254447.s003.tif]

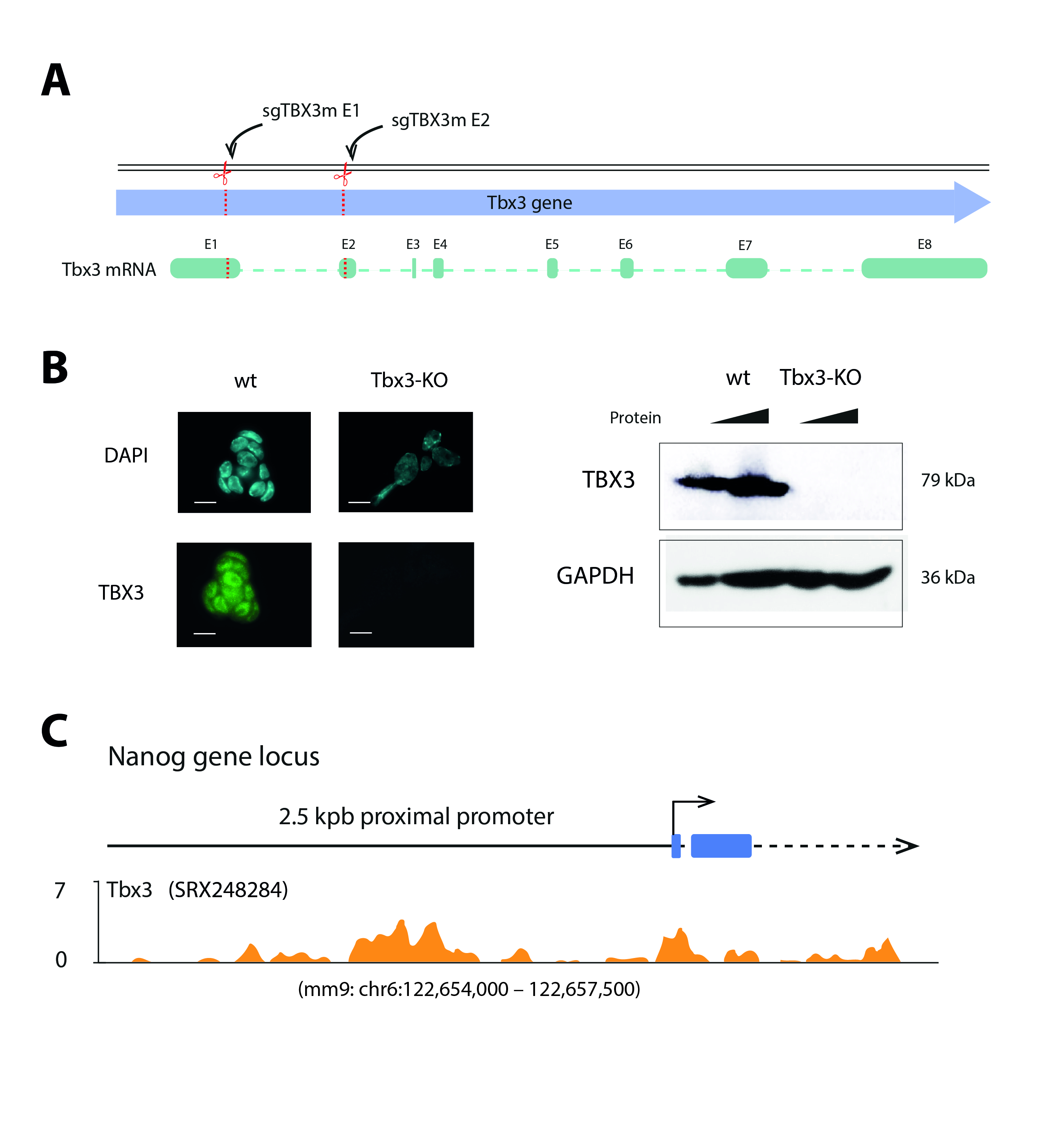

Supplement: S4 Fig — (A) Generation of Txb3 knockout (KO) ES cell line: Diagram of the Tbx3 gene (violet), mRNA (green) and the CRISPR-guided cleavage sites (red). CRISPR sgRNAs sgTBX3m E1 and E2 targeted the first and second coding exons, respectively [30]. The illustration was prepared using SnapGene Viewer (GSL Biotech; available at snapgene.com). (B) Left panel shows representative images of TBX3 immunostaining for wt and Tbx3-KO ES cell lines. Scale bar: 10 μm. Right panel shows Western blot demonstrating the absence of Tbx3 protein in Tbx3-KO ES cell line. Full blots are available at S1 File. (C) Visualization of representative enrichment profile (reads per million) of the Tbx3 TF in the 2.5 kpb region of the Nanog genomic locus included in Nanog5P reporter. The results shown correspond to the analysis of public ChIP-seq data from experiments performed in ES cells (Chip Atlas database: http://chip-atlas.org) [34]. Data was visualized using the Integrative Genomics Viewer (IGV) software [35]. Full meta-data of analyzed datasets is available at S3 Table. (TIF) [file pone.0254447.s004.tif]
