# Supplementary material for: SUMO conjugation susceptibility of Akt/protein kinase B affects the expression of the pluripotency transcription factor Nanog in embryonic stem cells
Source: PLoS One. 2021 Jul 9;16(7):e0254447. doi: 10.1371/journal.pone.0254447 (PMC8270172; doi:10.1371/journal.pone.0254447)

S1 File

SUMO conjugation susceptibility of Akt/Protein kinase B affects the expression of the pluripotency transcription factor Nanog in embryonic stem cells

Marcos Francia, Martin Stortz, Camila Vazquez Echegaray, Camila Oses, Paula Verneri, María Victoria Petrone, Ayelen Toro, Ariel Waisman, Santiago Miriuka, María Soledad Cosentino, Valeria Levi, and Alejandra Guberman.

Full blots corresponding to S1B Fig

S1B Fig is composed from the two WB experiments shown below. Since the lane corresponding to the control untransfected cells in the blot of the right, presented low amounts of total protein evidenced by the low density of GAPDH band, we replaced this control by another blot from the same sample. This blot is shown at the left parallelly run with a reference sample (wt) to control similar levels of total proteins.

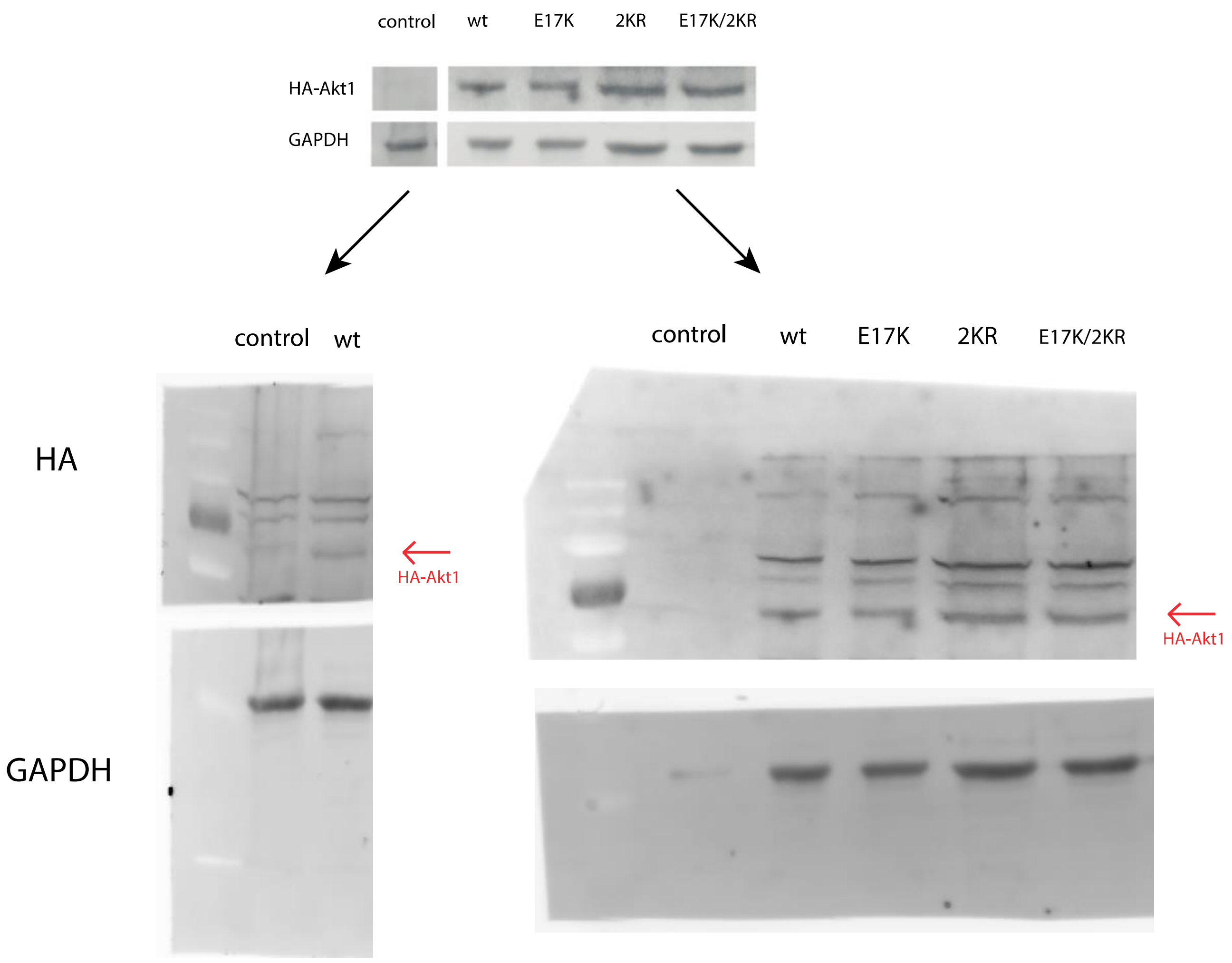

Identification of the HA-Akt1 band in blot from S1B Fig

Since anti-HA antibody revealed many unspecific bands, in order to confirm that the specific HA-Akt band was the expected one at 56 kDa, we performed the control shown below. We loaded protein extracts corresponding to the same samples in parallel lanes, run simultaneously in a single SDS-PAGE, transferred to a membrane, and cut it to blotting it with the Anti-Akt1 and Anti-HA antibodies. Anti-Akt1 recognizes both endogenous and transfected Akt, contrary, anti-HA reveals only the HA-tagged transfected Akt. This control allowed us to unequivocally identify the band corresponding

to transfected Akt. The protein extracts loaded at the left of the marker correspond to the same samples than those loaded at the right (control and HA-E17K), but each half of the membrane was blotted with the antibody indicated at the top of the panel.

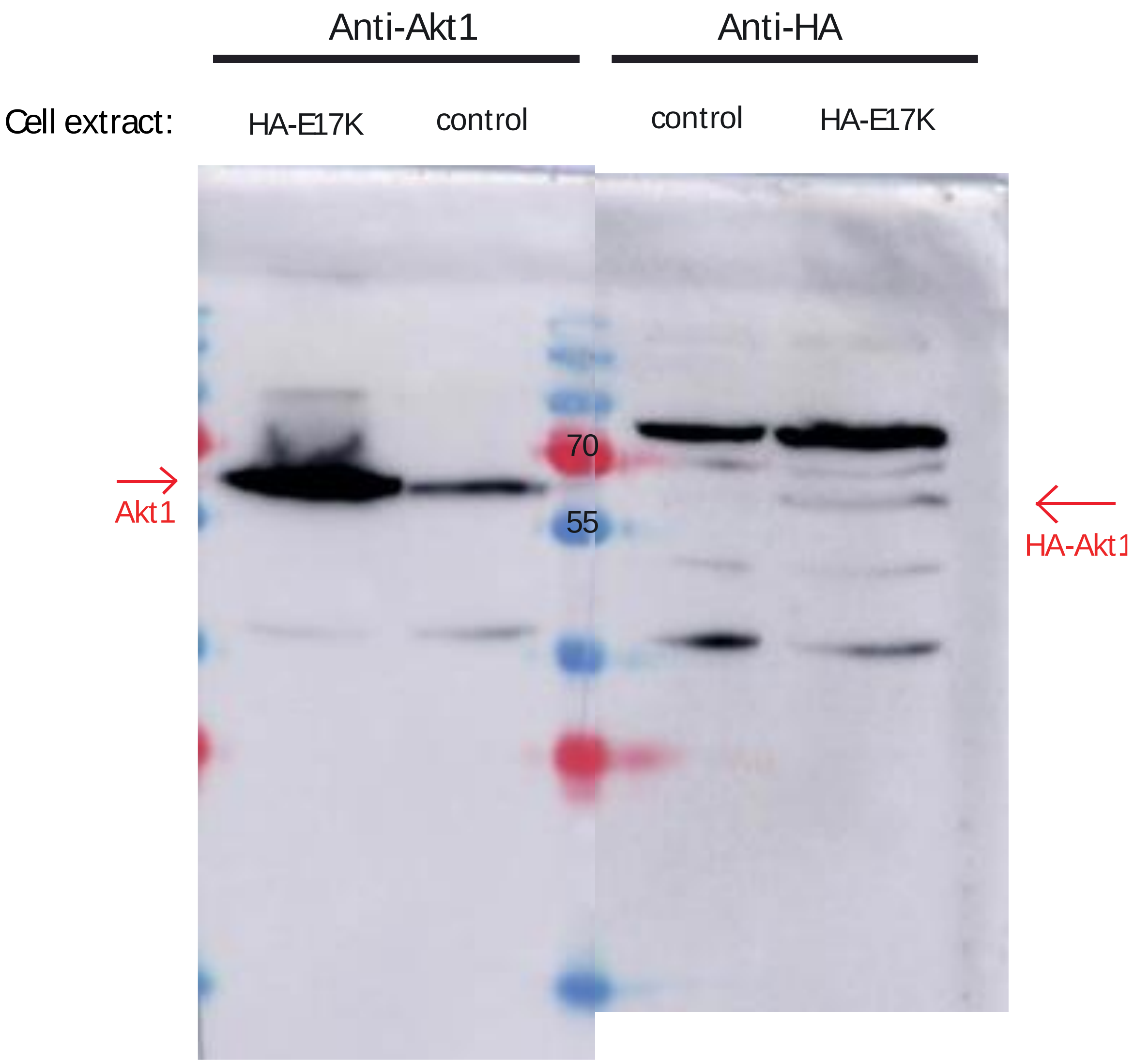

Full blots corresponding to S3B Fig

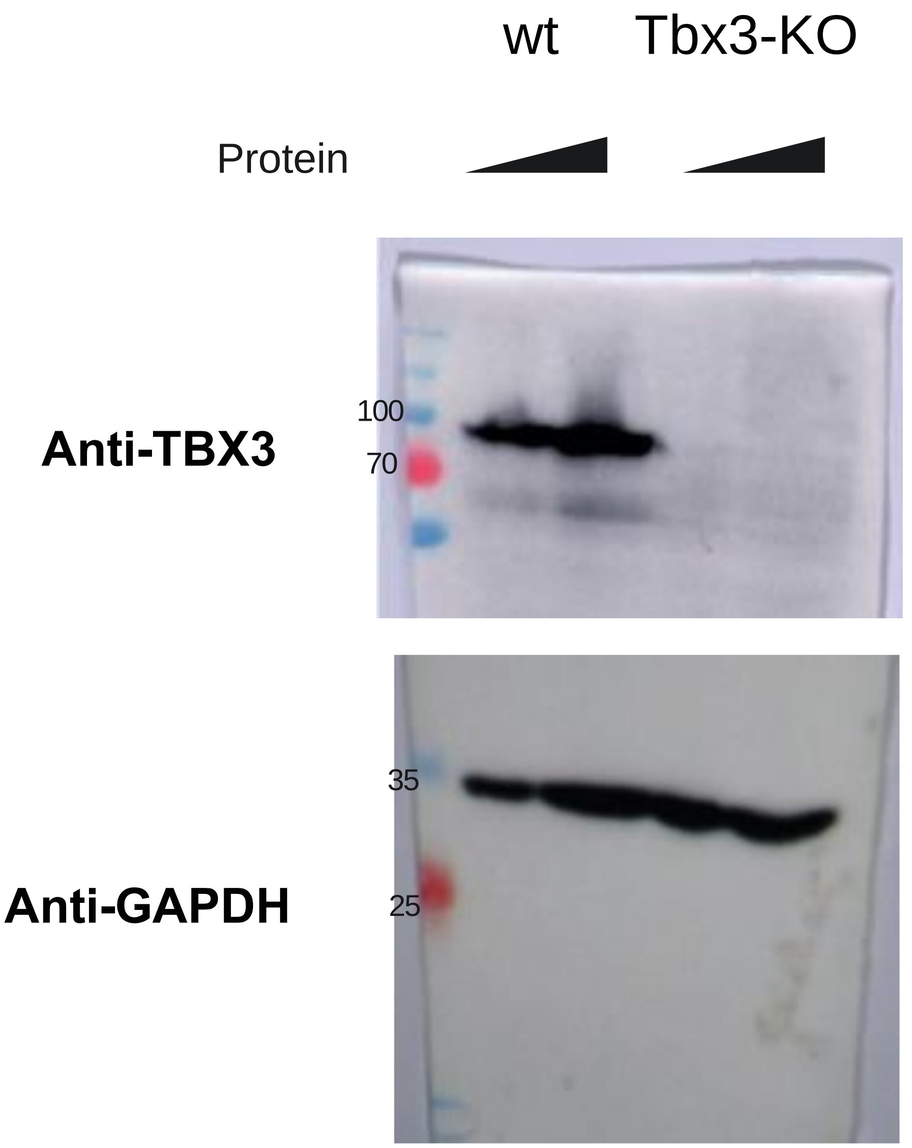

Supplement: S1 File — Uncropped blots of the western blot figures shown in this work. (PDF) [file pone.0254447.s008.pdf]
